# Five new sucrose esters from the whole plants of *Phyllanthus cochinchinensis*

**DOI:** 10.1007/s13659-013-0026-7

**Published:** 2013-04-13

**Authors:** Jian-Qiang Zhao, Yan-Ming Wang, Dong Wang, Chong-Ren Yang, Min Xu, Ying-Jun Zhang

**Affiliations:** 126 State Key Laboratory of Phytochemistry and Plant Resources in West China, Kunming Institute of Botany, Chinese Academy of Sciences, Kunming, 650201 China; 226 University of Chinese Academy of Sciences, Beijing, 100049 China

**Keywords:** *Phyllanthus cochinchinensis*, sucrose esters, secoiridoid glycosides

## Abstract

Chemical investigation of the whole plants of *Phyllanthus cochinchinensis* (Euphorbiaceae) led to the isolation of five new sucrose benzoyl esters, 3,6′-di-*O*-benzoylsucrose (**1**), 3,6′-di-*O*-benzoyl-2′-*O*-acetylsucrose (**2**), 3,6′-di-*O*-benzoyl-4′-*O*acetylsucrose (**3**), 3,6′-di-*O*-benzoyl-3′-*O*-acetylsucrose (**4**) and 3-*O*-benzoyl-6′-*O*-(*E*)-cinnamoylsucrose (**5**), together with two known secoiridoid glycosides, jasminoside (**6**) and jaslanceoside B (**7**). Their structures were established on the basis of detailed spectroscopic analysis and chemical method.

**Electronic Supplementary Material:**

Supplementary material is available for this article at 10.1007/s13659-013-0026-7 and is accessible for authorized users.

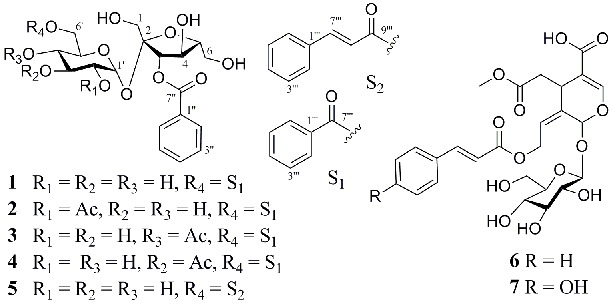

## Introduction

*Phyllanthus*, comprising about 600 species, is the largest genus in family Euphorbiaceaec, of which most species are important medicinal plants having been used for the treatment of infectious diseases. Previous chemical studies on this genus have revealed the occurrence of flavonoids, alkaloids, sesquiterpenoids, triterpenoids, lignans, and tannins.[1–5] Among them, some sesquiterpenoids exhibited potent antiviral activity against coxsackie virus B3 (CVB3),[4] and inhibition of the growth of murine P-388 lymphocytic leukemia cell line.[5]

*P. cochinchinensis*, a shrub up to 3 m height, is mainly growing in the montane sparse forests, forest margins, scrub on slopes, and wastelands of the southern part of China. It is also widely distributed in Cambodia, India, Laos, and Vietnam. So far, no chemical study was reported on this species. As a part of our continuing study on bioactive compounds from *Phyllanthus* species,[1,2,4] five new sucrose benzoyl esters, 3,6'-di-*O*-benzoylsucrose (**1**), 3,6'-di-*O*-benzoyl-2'-*O*-acetylsucrose (**2**), 3,6'-di-*O*-benzoyl-4'-*O*-acetylsucrose (**3**), 3,6'-di-*O*-benzoyl-3'-*O*-acetylsucrose (**4**) and 3-*O*-benzoyl-6'-*O*-(*E*)-cinnamoylsucrose (**5**), were isolated from the whole plants of *P. cochinchinensis*, together with two known secoiridoid glycosides, jasminoside (**6**) and jaslanceoside B (**7**). Their structures were established by means of MS and extensive NMR spectroscopic analysis and chemical method.

**Figure 1.** Structure of compounds **1**–**7**

## Results and Discussion

The air-dried and powdered whole plants of *P. cochinchinensis* were extracted with MeOH under reflux. Further column chromatography (CC) over Diaion HP20SS, Sephadex LH-20 and silica gel, followed with semi-preparative HPLC purification of the MeOH extract yielded five new compounds (**1**–**5**), together with two known ones. The known compounds were elucidated as jasminoside (**6**)[6] and jaslanceoside B (**7**)[7] by comparison of their spectroscopic data with reported literature values.

Compound **1** was isolated as a white amorphous powder. Its

*To whom correspondence should be addressed. E-mail: xumin@mail.kib.ac.cn (M. Xu); zhangyj@mail.kib.ac.cn (Y.J. Zhang)

**Table 1.** $^{1}$**H NMR spectroscopic data of compounds 1–5**

| Pos. | **1**[a] | **2**[b] | **3**[b] | **4**[b] | **5**[c] |
|---|---|---|---|---|---|
| 1 | 3.62, d (12.0); 3.67[d] | 3.45, d (11.8); 3.63, d (11.8) | 3.66, d (12.3); 3.71[d] | 3.63, d (12.5); 3.72[d] | 3.39[e]; 3.34[e] |
| 3 | 5.63, d (8.0) | 5.65, d (8.2) | 5.63, d (7.1) | 5.71, d (8.3) | 5.53, d (7.9) |
| 4 | 4.43, t (8.1) | 4.41, t (8.4) | 4.44, t (7.2) | 4.45, t (8.3) | 4.24, br. s |
| 5 | 3.96, m | 3.94, ddd (8.8, 6.6, 2.5) | 4.03, td (7.0, 3.5) | 4.00, ddd (8.4, 6.9, 2.7) | 3.85, td (7.6, 3.2) |
| 6 | 3.68[d]; 3.83[d] | 3.78[d]; 3.69, dd (12.1, 2.5) | 3.74, dd (12.0, 3.5); 3.84, dd (12.0, 6.8) | 3.71[d]; 3.86, m | 3.61, d (11.4); 3.73, br. s |
| 1' | 5.46, d (3.6) | 5.58, d (3.6) | 5.52, d (3.6) | 5.55, d (3.6) | 5.23, d (3.3) |
| 2' | 3.47[d] | 4.61, dd (10.1, 3.6) | 3.57, dd (9.8, 3.6) | 3.72[d] | 3.29[e] |
| 3' | 4.17, dd (9.9, 2.5) | 3.76[d] | 3.71[d] | 5.36, t (9.7) | 3.44[e] |
| 4' | 3.49[e] | 4.15, d (3.6) | 4.98[e] | 3.69, t (8.5) | 3.15, t (9.3) |
| 5' | 3.66, m | 3.56, t (9.5) | 4.32[d] | 4.29 ddd (10.0, 4.2, 2.0) | 4.03, t (8.4) |
| 6' | 4.50, dd (12.0, 4.5) | 4.50, dd (12.1, 2.1) | 4.45[d] | 4.55, dd (12.1, 4.3) | 4.18, dd (11.8, 7.1) |
| | 4.60, dd (12.0, 1.9) | 4.46, dd (12.1, 4.1) | 4.34, dd (12.2, 3.9) | 4.63, dd (12.1, 2.0) | 4.43, d (11.4) |
| 2",6" | 8.12, d (7.4) | 8.14, d (7.3) | 8.15, d (7.2) | 8.19, d (7.2) | 8.04, d (7.6) |
| 3",5" | 7.50, t (7.4) | 7.52, t (7.3) | 7.51, t (7.2) | 7.53, t (7.2) | 7.42[d] |
| 4" | 7.61, t (7.4) | 7.63, t (7.3) | 7.64, t (7.2) | 7.62, t (7.2) | 7.70[d] |
| 2"',6"' | 8.05, d (7.4) | 8.05, d (7.3) | 8.07, d (7.2) | 8.07, d (7.2) | 7.42[d] |
| 3"',5"' | 7.48, t (7.4) | 7.50, t (7.3) | 7.49, t (7.2) | 7.50, t (7.2) | 7.56, t (7.7) |
| 4"' | 7.61, t (7.4) | 7.61, t (7.3) | 7.62, t (7.2) | 7.60, t (7.2) | 7.70[d] |
| 7"' | | | | | 7.67, d (15.6) |
| 8"' | | | | | 6.68, d (15.6) |
| AcO | | 2.04, s | 2.06, s | 2.11, s | |

[a]Recorded in $CD_3OD$ at 500 MHz; [b]Recorded in $CD_3OD$ at 600 MHz; [c]Recorded in DMSO-$d_6$ at 600 MHz; [d]Overlapping $^{1}$H NMR signals; [e]Overlapped by solvent molecular formula was determined to be $C_{26}H_{30}O_{13}$, on the basis of HRESIMS (*m/z* 573.1578 $[M + Na]^+$). The IR spectrum showed the presence of hydroxyl (3432 $cm^{-1}$) and carbonyl (1721 $cm^{-1}$) groups. The $^{1}$H NMR spectrum of **1** (Table 1) displayed characteristic signals of two benzoyl groups [$\delta_H$ 8.12, 8.05 (each 2H, d, $J$ = 7.4 Hz), 7.50, 7.48 (each 2H, t, $J$ = 7.4 Hz), 7.61 (2H, t, $J$ = 7.4 Hz)]. The $^{13}$C NMR (DEPT) spectra of **1** (Table 2) gave 26 carbon signals, including 12 aromatic ($\delta_C$ 129.8–134.6) and two carbonyl carbons ($\delta_C$ 167.5 and 168.1) arising from two benzoyl units, and 12 oxygen-bearing alphatic carbons ($\delta_C$ 63.8–104.9) due to two hexosyl moieties. Alkaline hydrolysis of **1** with 0.5% NaOH in MeOH yielded sucrose ($[\alpha]_D^{16}$ + 28.5),[8] indicating that **1** is a bisbenzoyl sucrose ester. The $^{1}$H and $^{13}$C NMR signals of the sugar units were assigned unambiguously by $^{1}$H-$^{1}$H COSY, HSQC, and HMBC analysis (Tables 1 and 2). In the HMBC spectrum of **1**, a correlation of glucosyl anomeric proton at $\delta_H$ 5.46 (H-1') with the fructosyl C-2 ($\delta_C$ 104.9) confirmed the sucrose moiety in **1**. Furthermore, HMBC correlations of $\delta_H$ 5.63 (H-3) with $\delta_C$ 167.5 (C-7"), $\delta_C$ 74.3 (C-4) and $\delta_C$ 65.3 (C-1), and $\delta_H$ 4.50 (H-6'a) with $\delta_C$ 168.1 (C-7"') and $\delta_C$ 74.9 (C-5') indicated that the two benzoyl units were linked to C-3 and C-6' of sucrose moiety, respectively. Accordingly, compound **1** was determined to be 3,6'-di-*O*-benzoylsucrose.

Compound **2**, a white amorphous powder, gave an $[M + Na]^+$ peak at *m/z* 615.1675 ($C_{28}H_{32}O_{14}Na$) in HRESIMS, which was 42 Da more than that of **1**. The $^{1}$H and $^{13}$C NMR spectra (Tables 1 and 2) of **2** showed high similarity to those of **1**, except for the appearance of an additional acetyl group [$\delta_H$ 2.04 (3H, s) and $\delta_C$ 172.7 and 21.1]. In the HMBC spectrum of **2**, the correlation of $\delta_H$ 4.61 (H-2') with the acetyl carbonyl carbon at $\delta_C$ 172.7 was observed, allowing the assignment of the acetyl group located at C-2' of sucrose moiety. This also resulted in the higher field shifted carbon resonances of C-1' and C-3' of **2** as compared with those of **1** (Table 2). Thus, the structure of **2** was established as 3,6'-di-*O*-benzoyl-2'-*O*-acetylsucrose.

Compounds **3** and **4** had the same molecular formula

**Figure 2.** Key HMBC correlations of **1**

$C_{28}H_{32}O_{14}$, on the basis of their HRESIMS (*m/z* 615.1692 and 615.1689 $[M + Na]^+$ for **3** and **4**, respectively), which exhibited the same molecular weight with that of **2**. The extensive comparison of 1D and 2D NMR data with those of compound **2** suggested that both **3** and **4** had the same 3,6'-di-*O*-benzoylsucrose skeleton, while the difference among **2**, **3** and **4** was the position of the acetyl group. The $^{1}$H and $^{13}$C NMR signals of compounds **3** and **4** could be assigned unambiguously by HSQC, and HMBC analysis (Tables 1 and 2), respectively. In the HMBC experiment, H-4' ($\delta_H$ 4.98) in **3** and H-3' ($\delta_H$ 5.36) in **4** were correlated with the acetyl carbonyl carbon at $\delta_C$ 172.0 (**3**) and $\delta_C$ 172. 8 (**4**), respectively, indicating that the acetyl groups in **3** and **4** were located at C-4' and C-3', respectively. Other HMBC correlations confirmed the structures of **3** and **4** as shown in Fig 1. Consequently, compounds **3** and **4** were determined to be 3,6'-di-*O*-benzoyl-4'-*O*-acetylsucrose (**3**) and 3,6'-di-*O*-benzoyl-3'-*O*-acetylsucrose (**4**), respectively.

Compound **5**, a white amorphous powder, possessed a molecular formula $C_{28}H_{32}O_{13}$, as deduced from the HRESIMS (*m/z* 599.1737 $[M + Na]^+$) and $^{13}$C NMR (DEPT) spectra. The $^{13}$C NMR spectrum of **5** (Table 2) exhibited the presence of 12 oxygen-bearing carbon signals relating to two hexosyl units, two carbonyl carbons at $\delta_C$ 165.1 and 166.4, in addition to 12 aromatic methines ($\delta_C$ 118 to 145) and two aromatic quaternary carbons at $\delta_C$ 129.8 and 134.1, suggesting the

**Table 2.** $^{13}$C NMR spectroscopic data of compounds **1**–**5**

| Pos. | **1**[a] | **2**[b] | **3**[b] | **4**[b] | **5**[c] |
|---|---|---|---|---|---|
| 1 | 65.3, CH$_2$ | 64.8, CH$_2$ | 65.5, CH$_2$ | 65.0, CH$_2$ | 63.2, CH$_2$ |
| 2 | 104.9, C | 105.4, C | 105.5, C | 104.9, C | 103.2, C |
| 3 | 80.3, CH | 79.3, CH | 80.6, CH | 80.4, CH | 78.1, CH |
| 4 | 74.3, CH | 74.0, CH | 74.8, CH | 74.3, CH | 73.0, CH |
| 5 | 84.3, CH | 84.2, CH | 85.0, CH | 84.3, CH | 83.1, CH |
| 6 | 63.8, CH$_2$ | 63.7, CH$_2$ | 63.7, CH$_2$ | 63.9, CH$_2$ | 62.6, CH$_2$ |
| 1' | 93.5, CH | 91.0, CH | 93.3, CH | 93.5, CH | 91.3, CH |
| 2' | 73.1, CH | 74.5, CH | 73.0, CH | 71.3, CH | 71.3, CH |
| 3' | 72.6, CH | 72.2, CH | 72.8, CH | 76.8, CH | 72.9, CH |
| 4' | 71.5, CH | 72.3, CH | 72.5, CH | 69.7, CH | 70.2, CH |
| 5' | 74.9, CH | 71.5, CH | 70.1, CH | 72.6, CH | 70.7, CH |
| 6' | 65.3, CH$_2$ | 65.0, CH$_2$ | 64.4, CH$_2$ | 65.0, CH$_2$ | 64.6, CH$_2$ |
| 1" | 131.4, C | 131.4, C | 131.3, C | 131.3, C | 129.8, C |
| 2",6" | 131.1, CH | 131.1, CH | 131.1, CH | 131.2, CH | 129.5, CH |
| 3",5" | 129.9, CH | 130.0, CH | 129.9, CH | 130.0, CH | 128.9, CH |
| 4" | 134.6, CH | 134.8, CH | 134.7, CH | 134.6, CH | 133.6, CH |
| 7" | 167.5, C | 167.6, C | 167.1, C | 167.8, C | 165.1, C |
| 1"' | 131.3, C | 131.0, C | 131.2, C | 131.3, C | 134.1, C |
| 2"',6"' | 130.8, CH | 130.8, CH | 130.0, CH | 130.8, CH | 128.5, CH |
| 3"',5"' | 129.8, CH | 129.8, CH | 129.8, CH | 129.8, CH | 129.0, CH |
| 4"' | 134.5, CH | 134.5, CH | 134.6, CH | 134.5, CH | 130.6, CH |
| 7"' | 168.1, C | 168.1, C | 167.8, C | 168.0, C | 144.8, CH |
| 8"' | | | | | 118.0, CH |
| 9"' | | | | | 166.4, C |
| AcO | | 21.1, CH$_3$ | 21.2, CH$_3$ | 21.3, CH$_3$ | |
| | | 172.7, C | 172.0, C | 172.8, C | |

[a]Recorded in CD$_3$OD at 125 MHz; [b]Recorded in CD$_3$OD at 150 MHz; [c]Recorded in DMSO-$d_6$ at 150 MHz.

presence of two mono-substituted benzene rings and one double bond. The $^1$H NMR spectrum (Table 1) of **5** showed the presence of two *trans*-coupled olefinic protons at $\delta_H$ 6.68 and 7.67 (each 1H, d, $J$ = 15.6 Hz)]. The above data revealed that compound **5** was also an analogue of **1**, except for the additional double bond with *trans* configuration. All the proton and carbon signals in **5** were assigned unambiguously by HSQC and HMBC analysis (Tables 1 and 2). In the HMBC spectrum of **5** (Figure 3), correlations of the olefinic proton at $\delta_H$ 6.68 (H-8"') with one aromatic quanternary carbon at $\delta_C$ 134.1 (C-1"') and another olefinic proton at $\delta_H$ 7.67 (H-7"') with one carbonyl carbon at $\delta_C$ 166.4 (C-9"') indicated the additional *trans* double bond belonging to a *trans* cinnamoyl moiety. The HMBC correlations of the glucosyl H-6' ($\delta_H$ 4.43 and 4.18) with C-9"' ($\delta_C$ 166.4) and glucosyl C-4' ($\delta_C$ 70.2) revealed the linkage of the (*E*)-cinnamoyl moiety with C-6'. Moreover, the HMBC correlations of the fructosyl H-3 ($\delta_H$ 5.53) with $\delta_C$ 165.1 (C-7"), 63.2 (C-1) and 73.0 (C-4) suggested the benzoyl unit located at C-3. Other HMBC correlations (Figure 3) further confirmed the structures of **5** as shown in Fig 1. Therefore, compound **5** was determined to be 3-*O*-benzoyl-6'-*O*-(*E*)-cinnamoylsucrose.

The isolated compounds were evaluated for their cytotoxicities against five human cancer cell lines (breast cancer MCF-7, hepatocellular carcinoma SMMC-7721, human myeloid leukemia HL-60, colon cancer SW480, and lung cancer A-549). All of them showed no cytotoxic activity against the five human cancer cell lines at a concentration of 40 $\mu$M.

**Figure 3.** Key HMBC correlations of **5**

## Experimental Section

**General Experimental Procedures.** Optical rotations were measured with a HORIBA SEPA-300 high-sensitive polarimeter. IR spectra were measured on a Bio-Rad FTS-135 series spectrometer. UV spectra were recorded on a Shimadzu UV2401A ultraviolet-visible spectrophotometer. ESIMS and HRESIMS were run on an API QSTAR Pular-1 spectrometer. NMR spectra measured in methanol-$d_4$ or DMSO-$d_6$ solution and recorded on a Bruker AV-400, DRX-500 or AV III-600 spectrometer, using TMS as an internal standard. Chemical shifts were reported in units of $\delta$ (ppm) and coupling constants ($J$) were expressed in Hz. Column chromatography (CC) were carried out over silica gel (200–300 mesh, Qingdao Haiyang Chemical Co., Ltd., Qingdao, China), Diaion HP20SS (Mitsubishi Chemical Industry, Ltd., Tokyo, Japan), and Sephadex LH-20 (25–100 $\mu$m, Pharmacia Fine Chemical Co., Ltd., Uppsala, Sweden). Pre-coated silica gel plates (Qingdao Haiyang Chemical Co., Ltd., Qingdao, China) were used for TLC. Detection was done under UV light (254 and 365 nm) and by spraying the plates with 10% sulfuric acid followed by heating. An Agilent series 1260 (Agilent Technologies) were used for HPLC. An Agilent ZORBAX SB-C18 column 5 $\mu$m 143 Å column (250 mm × 9.4 mm) were used for semi-preparative HPLC separations.

**Plant Material.** The whole plant of *P. cochinchinensis* was collected from Guangdong Province, China, on December 2011. A voucher specimen (KUN-1215860) was deposited at the State Key Laboratory of Phytochemistry and Plant

Resources in West China, Kunming Institute of Botany, Chinese Academy of Sciences, and was identified by Prof. Xiao-Min Fang.

**Extraction and Isolation.** The air-dried and powdered whole plants of *P. cochinchinensis* (3.2 kg) were extracted with MeOH (3 times, 3h each time) under reflux at 60 °C. Evaporation of the solvent under vacuum gave a residue (270 g), which was suspended in water and then extracted sequentially with chloroform and butanol. The butanol extract (90 g) was chromatographed on Diaion HP20SS eluting with a gradient of MeOH-$H_2O$ (1:9 → 9:1, finally MeOH), to give 6 fractions F1–F6. F6 (16 g) was subjected to Sephadex LH-20 CC eluted with a gradient MeOH-$H_2O$ (1:9 → 6:4) to attain 3 fractions (F601–F603). F602 was fractionated through silica gel CC, using $CHCl_3$-MeOH-$H_2O$ (90:10:1 and 80:20:2) as solvent to afford 4 subfractions (F0201–F0204). F0201 (60 mg) was purified by semi-preparative HPLC (16%, MeCN-$H_2O$) to furnish **6** ($t_R$ 20 min, 17 mg). F0202 was fractioned through semi-preparative HPLC (17%, MeCN-$H_2O$) to afford two fractions at 15 min and at 18 min. The fraction at 15 min was purified through semi-preparative HPLC (17%, MeCN-$H_2O$) to attain **5** ($t_R$ 14 min, 2 mg), and the other fraction at 18 min was further purified by semi-preparative HPLC (18%, MeCN-$H_2O$) to afford **1** ($t_R$ 15 min, 13 mg), **2** ($t_R$ 16 min, 4 mg), **3** ($t_R$ 17 min, 6 mg), and **4** ($t_R$ 18 min, 3 mg). F5 (2.0 g) was chromatographed through Sephadex LH-20 CC eluted with a gradient MeOH-$H_2O$ (1:9 → 6:4) to attain 2 fractions (F501-F502). F502 (315 mg) was purified through silica gel CC, using $CHCl_3$-MeOH-$H_2O$ (90:10:1 and 80:20:2) as solvent, and then subjected to semi-preparative HPLC (18%, MeCN-$H_2O$) to afford **7** (88 mg).

**3,6'-Di-*O*-benzoylsucrose (1):** white amorphous powder; $[\alpha]_D^{22}$ + 30.2 (*c* 0.18, MeOH). UV (MeOH) $\lambda_{max}$ (log $\varepsilon$) 200 (4.24), 229 (4.33), 273 (3.26) nm; IR (KBr) $\nu_{max}$ 3432, 2924, 1721, 1603, 1453, 1379, 1279, 1124, 1070, 994 $cm^{-1}$; $^1H$ and $^{13}C$ NMR data, see Tables 1 and 2; negative ESIMS *m/z* 585 $[M + Cl]^-$; positive HRESIMS *m/z* 573.1578 $[M + Na]^+$ (calcd for $C_{26}H_{30}O_{13}Na$, 573.1578).

**3,6'-Di-*O*-benzoyl-2'-*O*-acetylsucrose (2):** white amorphous powder; $[\alpha]_D^{22}$ + 30.9 (*c* 0.08, MeOH). UV (MeOH) $\lambda_{max}$ (log $\varepsilon$) 200 (4.28), 229 (4.36), 273 (3.31) nm; IR (KBr) $\nu_{max}$ 3427, 2924, 2854, 1721, 1631, 1603, 1453, 1278, 1123, 1070, 1052, 994 $cm^{-1}$; $^1H$ and $^{13}C$ NMR data, see Tables 1 and 2; negative ESIMS *m/z* 627 $[M + Cl]^-$; positive HRESIMS *m/z* 615.1675 $[M + Na]^+$ (calcd for $C_{28}H_{32}O_{14}Na$, 615.1684).

**3,6'-Di-*O*-benzoyl-4'-*O*-acetylsucrose (3):** white amorphous powder; $[\alpha]_D^{22}$ + 26.0 (*c* 0.17, MeOH). UV (MeOH) $\lambda_{max}$ (log $\varepsilon$) 200 (4.06), 229 (4.18), 273 (3.09) nm; IR (KBr) $\nu_{max}$ 3425, 2926, 1721, 1603, 1453, 1376, 1279, 1119, 1052, 939 $cm^{-1}$; $^1H$ and $^{13}C$ NMR data, see Tables 1 and 2; positive ESIMS 615 $[M + Na]^+$; positive HRESIMS *m/z* 615.1692 $[M + Na]^+$ (calcd for $C_{28}H_{32}O_{14}Na$, 615.1684).

**3,6'-Di-*O*-benzoyl-3'-*O*-acetylsucrose (4):** white amorphous powder; $[\alpha]_D^{22}$ + 29.3 (*c* 0.07, MeOH). UV (MeOH) $\lambda_{max}$ (log $\varepsilon$) 200 (4.23), 229 (4.28), 273 (3.20) nm; IR (KBr) $\nu_{max}$ 3427, 2925, 1721, 1603, 1452, 1376, 1279, 1120, 1054, 1000 $cm^{-1}$; $^1H$ and $^{13}C$ NMR data, see Tables 1 and 2; positive ESIMS 615 $[M + Na]^+$; positive HRESIMS *m/z* 615.1689 $[M + Na]^+$ (calcd for $C_{28}H_{32}O_{14}Na$, 615.1684).

**3-*O*-Benzoyl-6'-*O*-(*E*)-cinnamoylsucrose (5):** white amorphous powder; $[\alpha]_D^{22}$ + 25.6 (*c* 0.07, MeOH). UV (MeOH) $\lambda_{max}$ (log $\varepsilon$) 201 (4.28), 220 (4.26), 278 (4.21) nm; IR (KBr) $\nu_{max}$ 3439, 2924, 1709, 1635, 1452, 1281, 1179, 1071, 1056, 1000 $cm^{-1}$; $^1H$ and $^{13}C$ NMR data, see Tables 1 and 2; negative ESIMS *m/z* 611 $[M + Cl]^-$; positive HRESIMS *m/z* 599.1737 $[M + Na]^+$ (calcd for $C_{28}H_{32}O_{13}Na$, 599.1737).

**Alkaline Hydrolysis of 1**. A mixture of **1** (6 mg), 0.5% NaOH (0.5 mL), and MeOH (3 mL) was stirred at room temperature for 6 h. The reaction mixture was neutralized with 1 N HCl and extracted with $CHCl_3$ (3 × 10 mL). The aqueous layer was applied to silica gel CC, eluting with $CHCl_3$-MeOH-$H_2O$ (6:4:1), to give sucrose (1.0 mg): $[\alpha]_D^{16}$ + 28.5 (*c* 0.8, $H_2O$); positive ESIMS *m/z* 365 $[M + Na]^+$. The alkaline hydrolysis of **2**–**5** was not preformed due to the limited amount of samples. The disaccharides of **2**–**5** were determined to be sucrose on the basis of biogenetic arguments of **1**.[8]

**Cytotoxicity Assay.** Five human cancer cell lines, human myeloid leukemia HL-60, hepatocellular carcinoma SMMC-7721, lung cancer A-549 cells, breast cancer MCF-7, and colon cancer SW480, were used in the cytotoxic assay. All the cells were cultured in RPMI-1640 or DMEM medium (Hyclone, USA), supplemented with 10% fetal bovine serum (Hyclone, USA). The cytotoxicity assay was performed according to the MTT (3-(4,5-dimethylthiazol-2-yl)-2,5-diphenyltetrazolium bromide) method in 96-well microplates.[9] Briefly, adherent cells (100 $\mu$L) was seeded into each well of 96-well cell culture plates and allowed to adhere for 12 h before drug addition, while suspended cells were seeded just before drug addition with an initial density of 0.5 × $10^5$–1 × $10^5$ cells/mL. Each tumor cell line was exposed to the test compound dissolved in DMSO in triplicates for 48 h at 37 °C, with DDP and taxol (Sigma, USA) as positive controls. Then, MTT (50 $\mu$L) was added to each well, and the tumor cells were incubated for another 4 h at 37 °C. After the supernatant liquor was removed, SDS (200 $\mu$L) was added to each well. The optical density was measured at 595 nm on a microplate reader. Cell viability was detected and a cell growth curve was graphed. $IC_{50}$ values were calculated by Reed and Muench's method.[10]

## Electronic Supplementary Material

Supplementary material is available in the online version of this article at http://dx.doi.org/ 10.1007/s13659-013-0026-7 and is accessible for authorized users.

## Acknowledgments

The authors are grateful to the members of the Analytical Group in State Key Laboratory of Phytochemistry and Plant Resources in West China, Kunming Institute of Botany, for measurements of all spectra. We would like to acknowledge

Prof. Yan Li for cytotoxicity assay. This work was supported by the NSFC 21002105, the 973 Program of Ministry of Science and Technology of China (2011CB915503), the Fourteenth Batch Candidates of the Young Academic Leaders of Yunnan Province (Min XU, 2011CI044) and by West Light Foundation of the Chinese Academy of Sciences.

**Open Access** This article is distributed under the terms of the Creative Commons Attribution License which permits any use, distribution, and reproduction in any medium, provided the original author(s) and source are credited.

## Electronic supplementary material

Supplementary material, approximately 1.08 MB.
