# Supplementary material for: Five new sucrose esters from the whole plants of Phyllanthus cochinchinensis
Source: Nat Prod Bioprospect. 2013 Apr 13;3(2):61–5. doi: 10.1007/s13659-013-0026-7 (PMC4131661; doi:10.1007/s13659-013-0026-7)
Supplement: Supplementary file 1 — Supplementary material, approximately 1.08 MB. [file 13659_2013_26_MOESM1_ESM.pdf]

**Electronic Supplementary Material**

# Five new sucrose esters from the whole plants of *Phyllanthus cochinchinensis*

Jian-Qiang ZHAO,[a,b,†] Yan-Ming WANG,[a,b,†] Dong WANG,[a] Chong-Ren YANG,[a] Min XU,[a,*] and Ying-Jun ZHANG[a,*]

[a]State Key Laboratory of Phytochemistry and Plant Resources in West China, Kunming Institute of Botany, Chinese Academy of Sciences, Kunming 650201, China

[b]University of Chinese Academy of Sciences, Beijing 100049, China

[†]These authors contributed equally to this work.

Received 21 March 2013; Accepted 5 April 2013

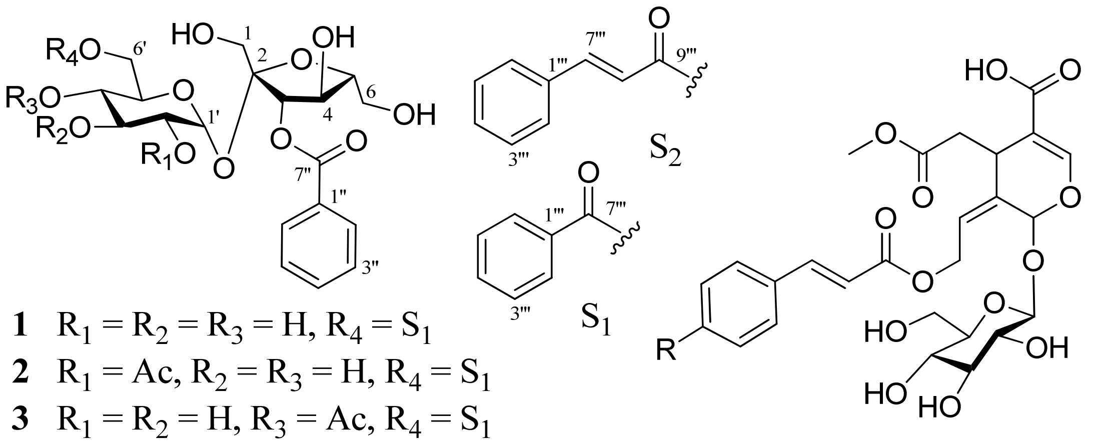


**1** $R_1 = R_2 = R_3 = H$, $R_4 = S_1$
**2** $R_1 = Ac$, $R_2 = R_3 = H$, $R_4 = S_1$
**3** $R_1 = R_2 = H$, $R_3 = Ac$, $R_4 = S_1$
**4** $R_1 = R_3 = H$, $R_2 = Ac$, $R_4 = S_1$
**5** $R_1 = R_2 = R_3 = H$, $R_4 = S_2$

**6** R = H
**7** R = OH

Structures of compounds **1**–**7**

*To whom correspondence should be addressed. E-mail: xumin@mail.kib.ac.cn (M. Xu); zhangyj@mail.kib.ac.cn (Y.J. Zhang)

S2 $^{1}$H NMR spectrum of 3,6'-di-*O*-benzoylsucrose (**1**) in methanol-$d_4$
S3 $^{13}$C NMR (DEPT) spectra of 3,6'-di-*O*-benzoylsucrose (**1**) in methanol-$d_4$
S4 HSQC spectrum of 3,6'-di-*O*-benzoylsucrose (**1**) in methanol-$d_4$
S5 $^{1}$H-$^{1}$H COSY spectrum of 3,6'-di-*O*-benzoylsucrose (**1**) in methanol-$d_4$
S6 HMBC spectrum of 3,6'-di-*O*-benzoylsucrose (**1**) in methanol-$d_4$
S7 Optical rotation spectrum of 3,6'-di-*O*-benzoylsucrose (**1**) in methanol-$d_4$
S8 $^{1}$H NMR spectrum of 3,6'-di-*O*-benzoyl-2'-*O*-acetylsucrose (**2**) in methanol-$d_4$
S9 $^{13}$C NMR (DEPT) spectra of 3,6'-di-*O*-benzoyl-2'-*O*-acetylsucrose (**2**) in methanol-$d_4$
S10 HSQC spectrum of 3,6'-di-*O*-benzoyl-2'-*O*-acetylsucrose (**2**) in methanol-$d_4$
S11 HMBC spectrum of 3,6'-di-*O*-benzoyl-2'-*O*-acetylsucrose (**2**) in methanol-$d_4$
S12 Optical rotation spectrum of 3,6'-di-*O*-benzoyl-2'-*O*-acetylsucrose (**2**) in methanol-$d_4$
S13 $^{1}$H NMR spectrum of 3,6'-di-*O*-benzoyl-4'-*O*-acetylsucrose (**3**) in methanol-$d_4$
S14 $^{13}$C NMR (DEPT) spectra of 3,6'-di-*O*-benzoyl-4'-*O*-acetylsucrose (**3**) in methanol-$d_4$
S15 HSQC spectrum of 3,6'-di-*O*-benzoyl-4'-*O*-acetylsucrose (**3**) in methanol-$d_4$
S16 HMBC spectrum of 3,6'-di-*O*-benzoyl-4'-*O*-acetylsucrose (**3**) in methanol-$d_4$
S17 Optical rotation spectrum of 3,6'-di-*O*-benzoyl-4'-*O*-acetylsucrose (**3**) in methanol-$d_4$
S18 $^{1}$H NMR spectrum of 3,6'-di-*O*-benzoyl-3'-*O*-acetylsucrose (**4**) in methanol-$d_4$
S19 $^{13}$C NMR (DEPT) spectra of 3,6'-di-*O*-benzoyl-3'-*O*-acetylsucrose (**4**) in methanol-$d_4$
S20 HSQC spectrum of 3,6'-di-*O*-benzoyl-3'-*O*-acetylsucrose (**4**) in methanol-$d_4$
S21 HMBC spectrum of 3,6'-di-*O*-benzoyl-3'-*O*-acetylsucrose (**4**) in methanol-$d_4$
S22 Optical rotation spectrum of 3,6'-di-*O*-benzoyl-3'-*O*-acetylsucrose (**4**) in methanol-$d_4$
S23 $^{1}$H NMR spectrum of 3-*O*-benzoyl-6'-*O*-(*E*)-cinnamoylsucrose (**5**) in DMSO-$d_6$
S24 $^{13}$C NMR (DEPT) spectra of 3-*O*-benzoyl-6'-*O*-(*E*)-cinnamoylsucrose (**5**) in DMSO-$d_6$
S25 HSQC spectrum of 3-*O*-benzoyl-6'-*O*-(*E*)-cinnamoylsucrose (**5**) in DMSO-$d_6$
S26 HMBC spectrum of 3-*O*-benzoyl-6'-*O*-(*E*)-cinnamoylsucrose (**5**) in DMSO-$d_6$
S27 Optical rotation spectrum of 3-*O*-benzoyl-6'-*O*-(*E*)-cinnamoylsucrose (**5**) in methanol-$d_4$

**S2** $^1$H NMR spectrum of 3,6'-di-*O*-benzoylsucrose (1) in methanol-$d_4$

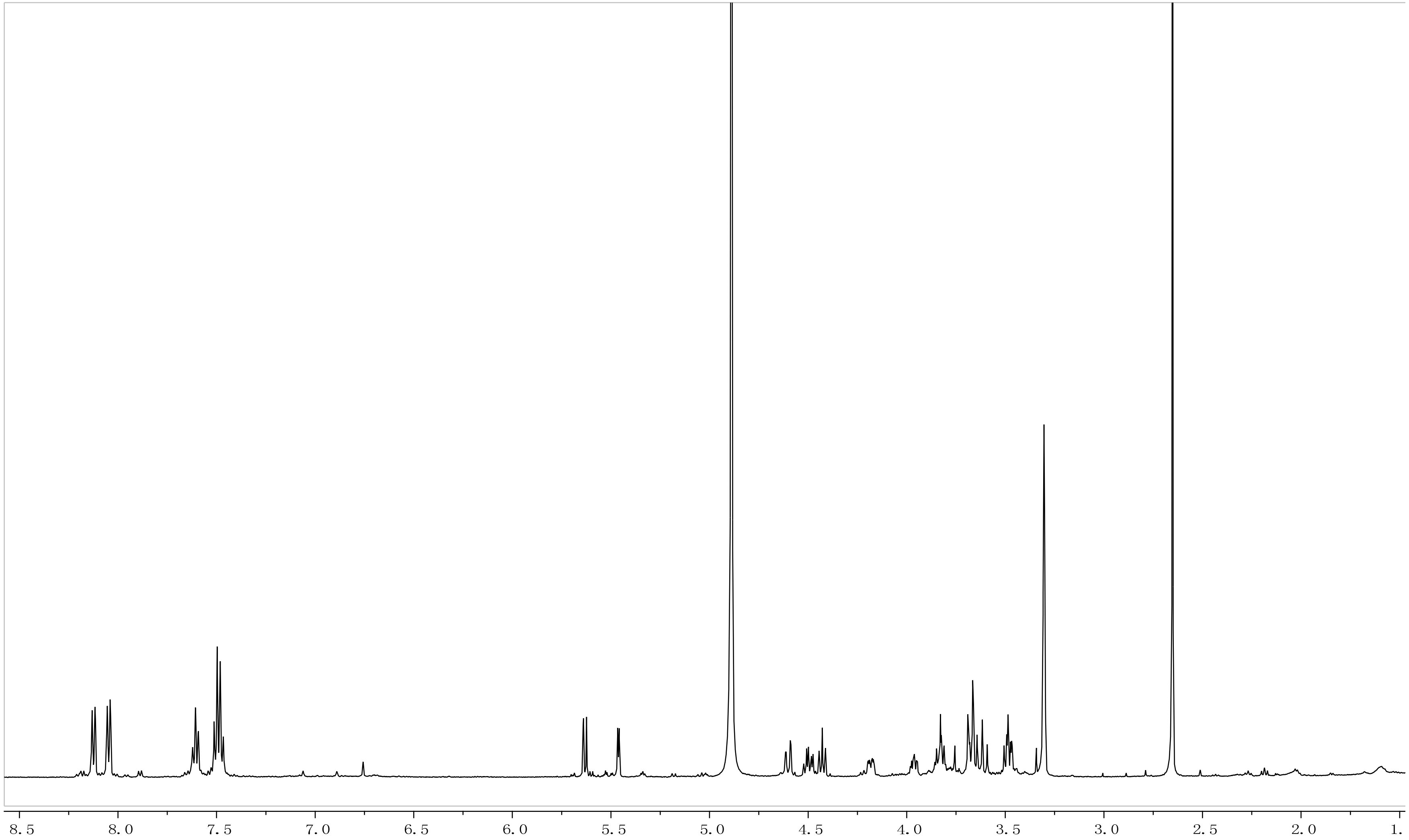

**S3 $^{13}$C NMR (DEPT) spectra of 3,6'-di-*O*-benzoylsucrose (1) in methanol-*d$_4$***

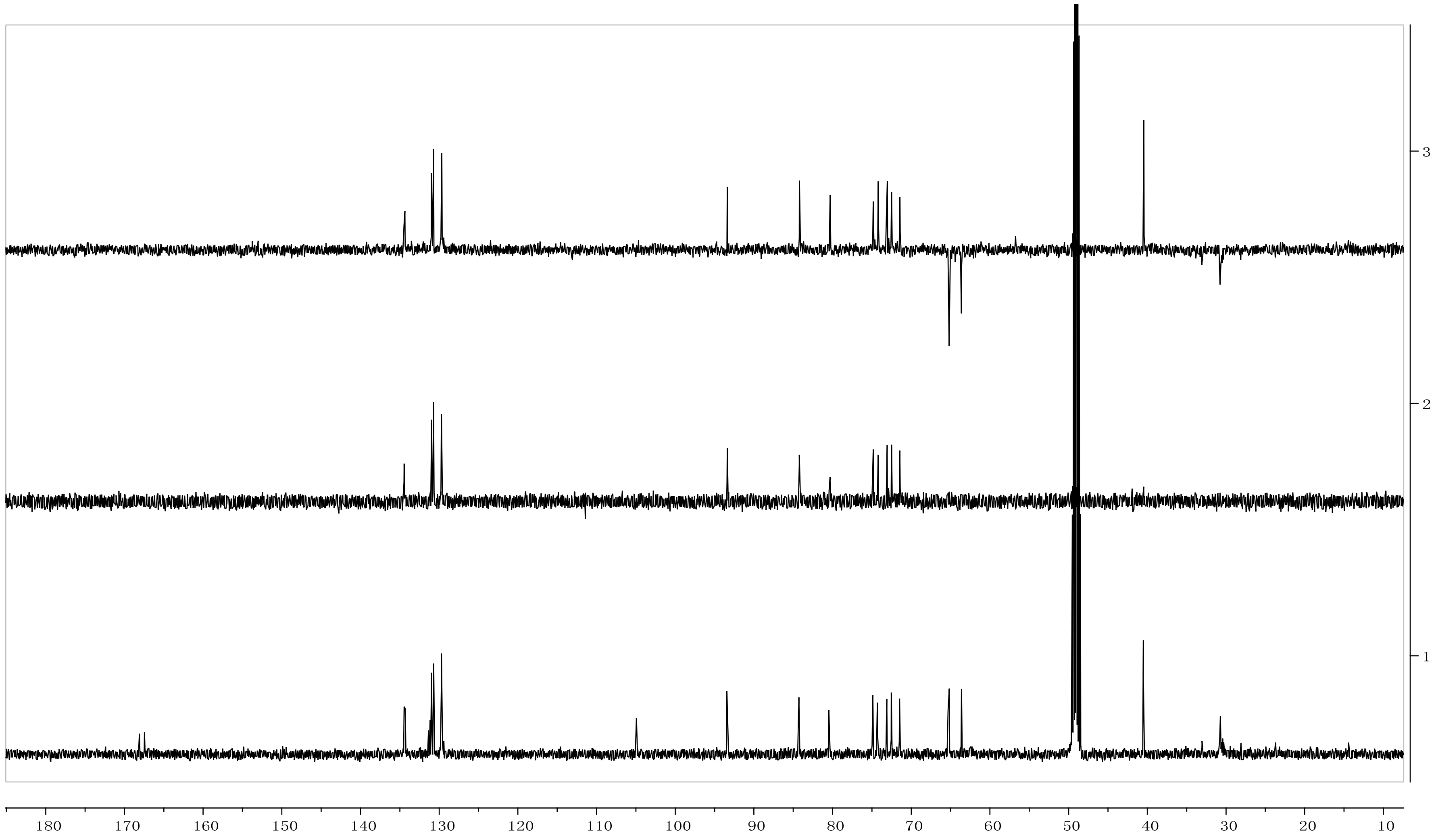

**S4 HSQC spectrum of 3,6'-di-*O*-benzoylsucrose (1) in methanol-$d_4$**

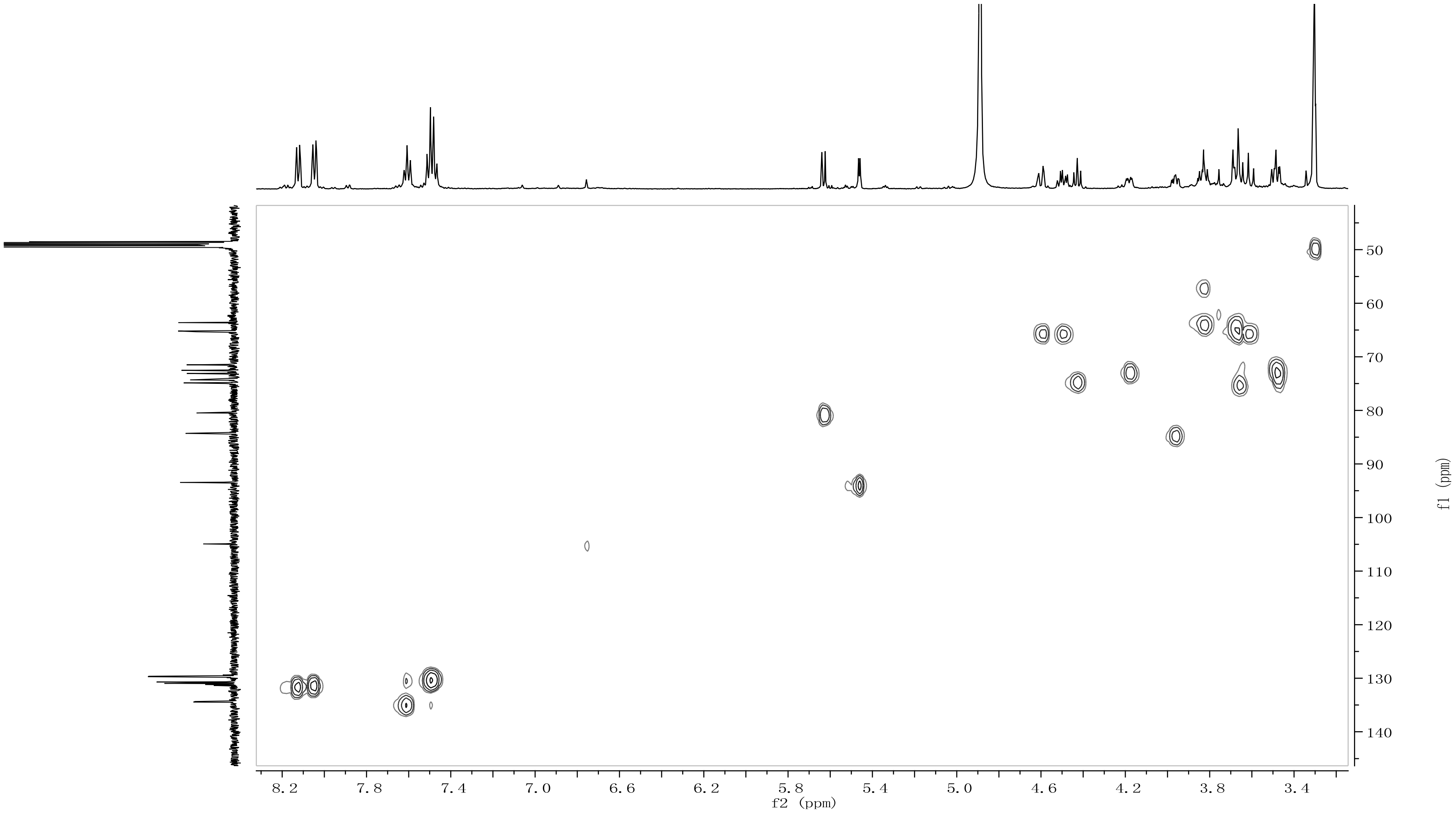

**S5 $^{1}H$-$^{1}H$ COSY spectrum for 3,6'-di-*O*-benzoylsucrose (1) in methanol-*d*$_4$**

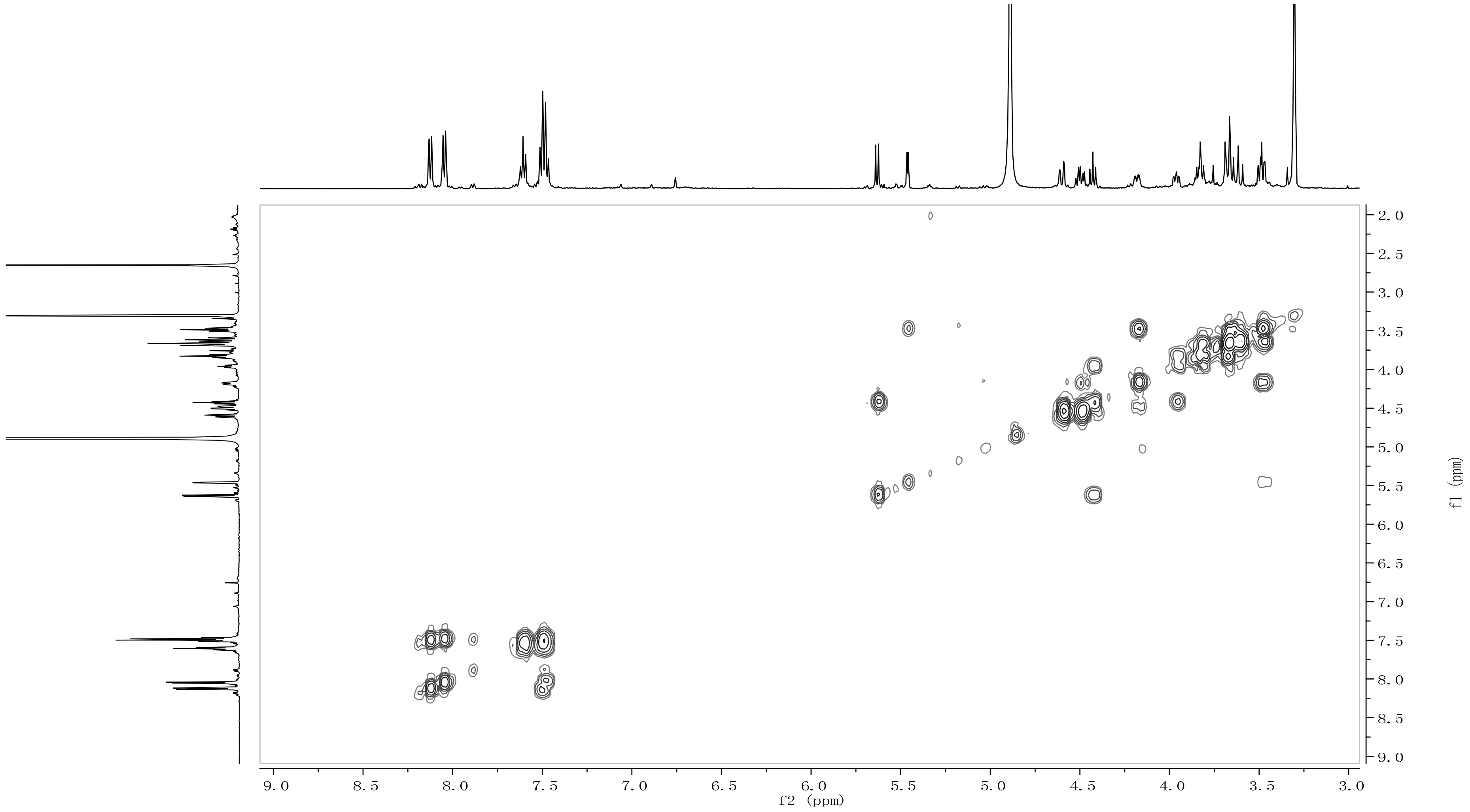

**S6 HMBC spectrum of 3,6'-di-*O*-benzoylsucrose (1) in methanol-$d_4$**

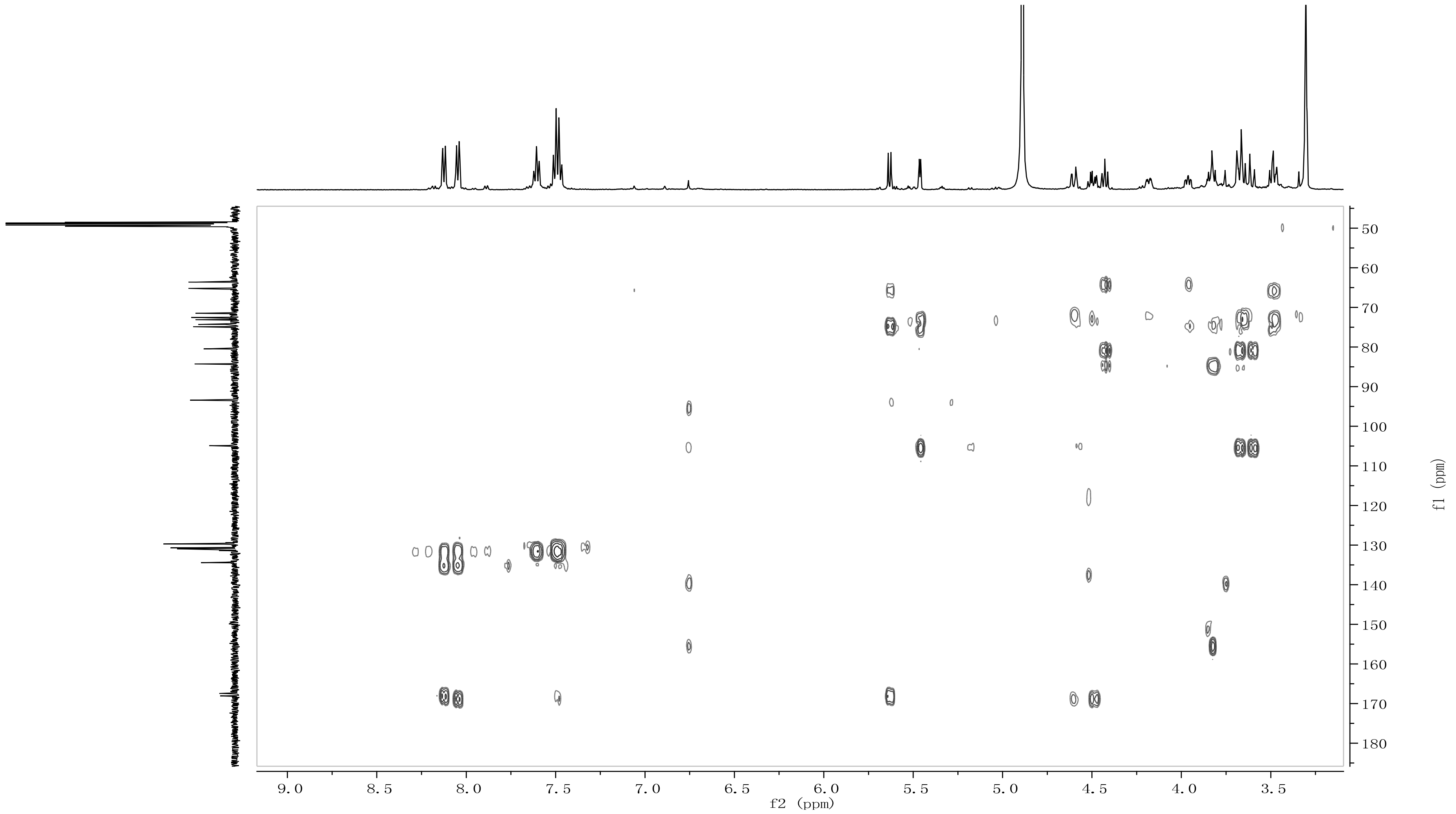

## S7 Optical rotation spectrum of 3,6'-di-*O*-benzoylsucrose (1) in methanol-$d_4$

Optical rotation measurement

Model : P-1020 (A060460638)

| No. | Sample | Mode | Data | Monitor<br>Blank | Temp.<br>Cell<br>Temp Point | Date<br>Comment<br>Sample Name | Light<br>Filter<br>Operator | Cycle Time<br>Integ Time |
|---|---|---|---|---|---|---|---|---|
| No.1 | 11 (1/3) | Sp.Rot | 30.2220 | 0.0272 | 22.3 | Mon Apr 01 14:35:15 2013 | Na | 2 sec |
| | | | | 0.0000 | 50.00 | 0.00180g/mlMeOH | 589nm | 10 sec |
| | | | | | Cell | YB622EA | | |
| No.2 | 11 (2/3) | Sp.Rot | 30.6670 | 0.0276 | 22.3 | Mon Apr 01 14:35:29 2013 | Na | 2 sec |
| | | | | 0.0000 | 50.00 | 0.00180g/mlMeOH | 589nm | 10 sec |
| | | | | | Cell | YB622EA | | |
| No.3 | 11 (3/3) | Sp.Rot | 29.7780 | 0.0268 | 22.3 | Mon Apr 01 14:35:42 2013 | Na | 2 sec |
| | | | | 0.0000 | 50.00 | 0.00180g/mlMeOH | 589nm | 10 sec |
| | | | | | Cell | YB622EA | | |

+30.2222°

**S8** $^1$H NMR spectrum of 3,6'-di-*O*-benzoyl-2'-*O*-acetylsucrose (2) in methanol-$d_4$

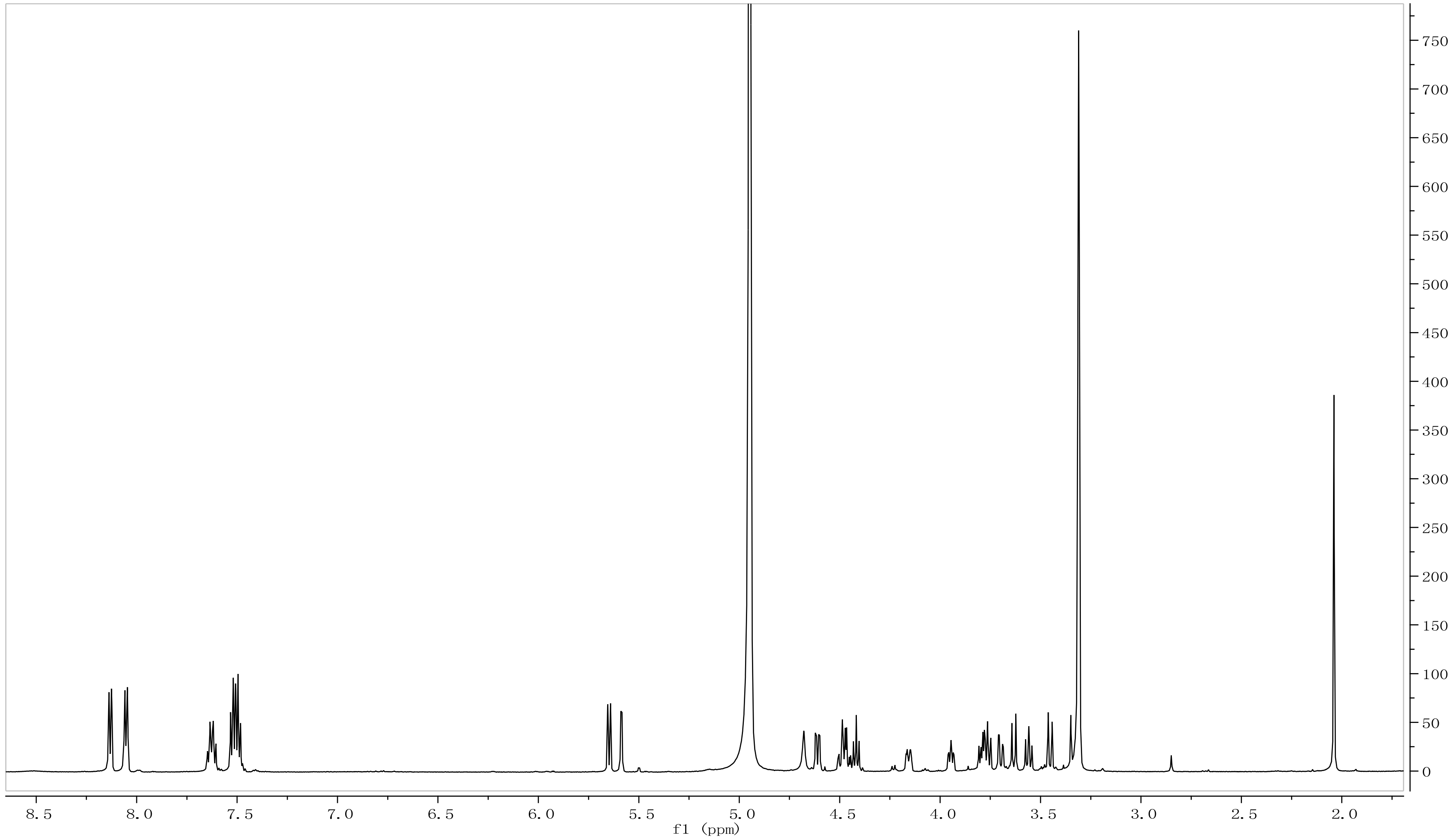

**S9 $^{13}$C NMR (DEPT) spectra of 3,6'-di-*O*-benzoyl-2'-*O*-acetylsucrose (2) in methanol-$d_4$**

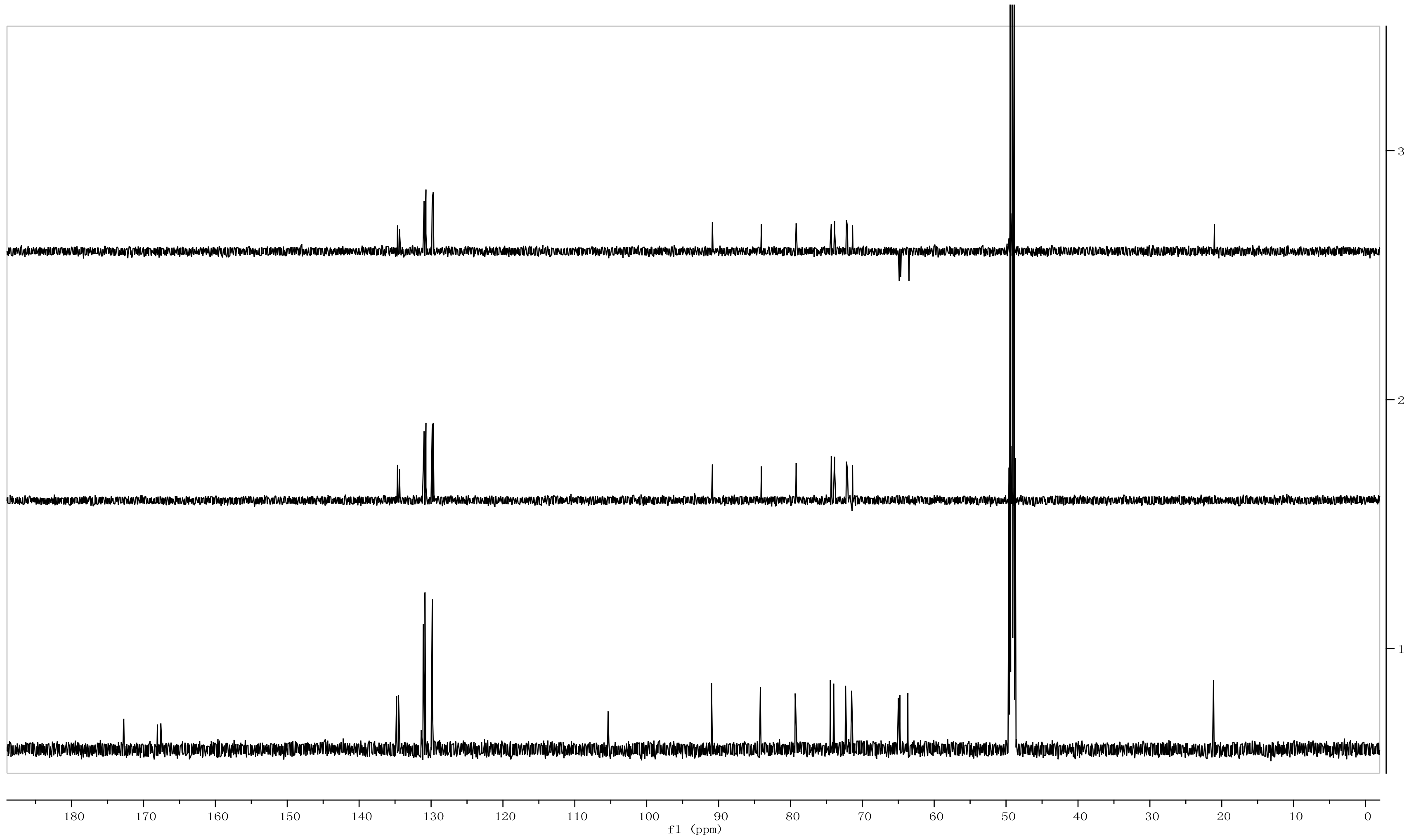

**S10 HSQC spectrum of 3,6'-di-*O*-benzoyl-2'-*O*-acetylsucrose (2) in methanol-$d_4$**

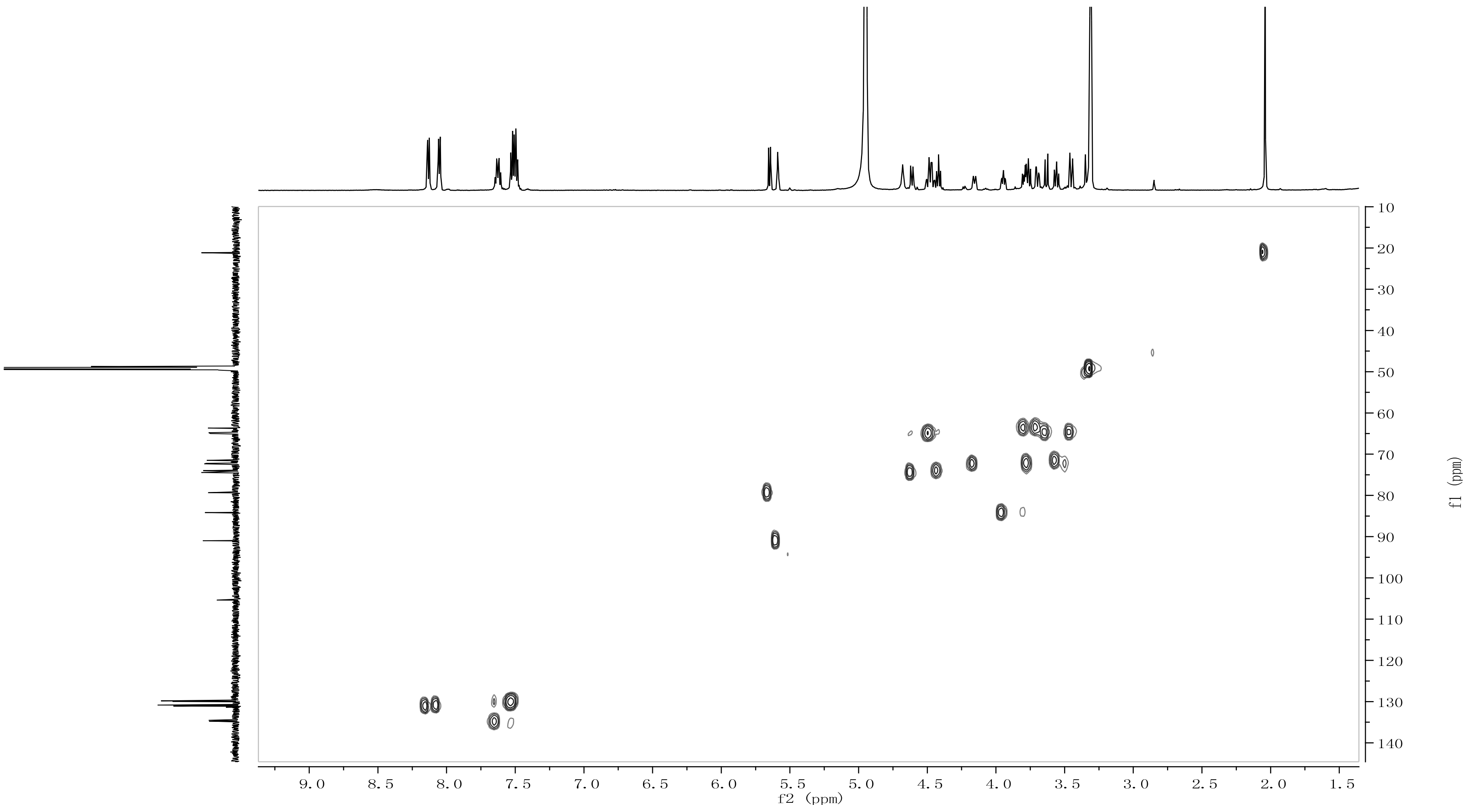

**S11 HMBC spectrum of 3,6'-di-*O*-benzoyl-2'-*O*-acetylsucrose (2) in methanol-$d_4$**

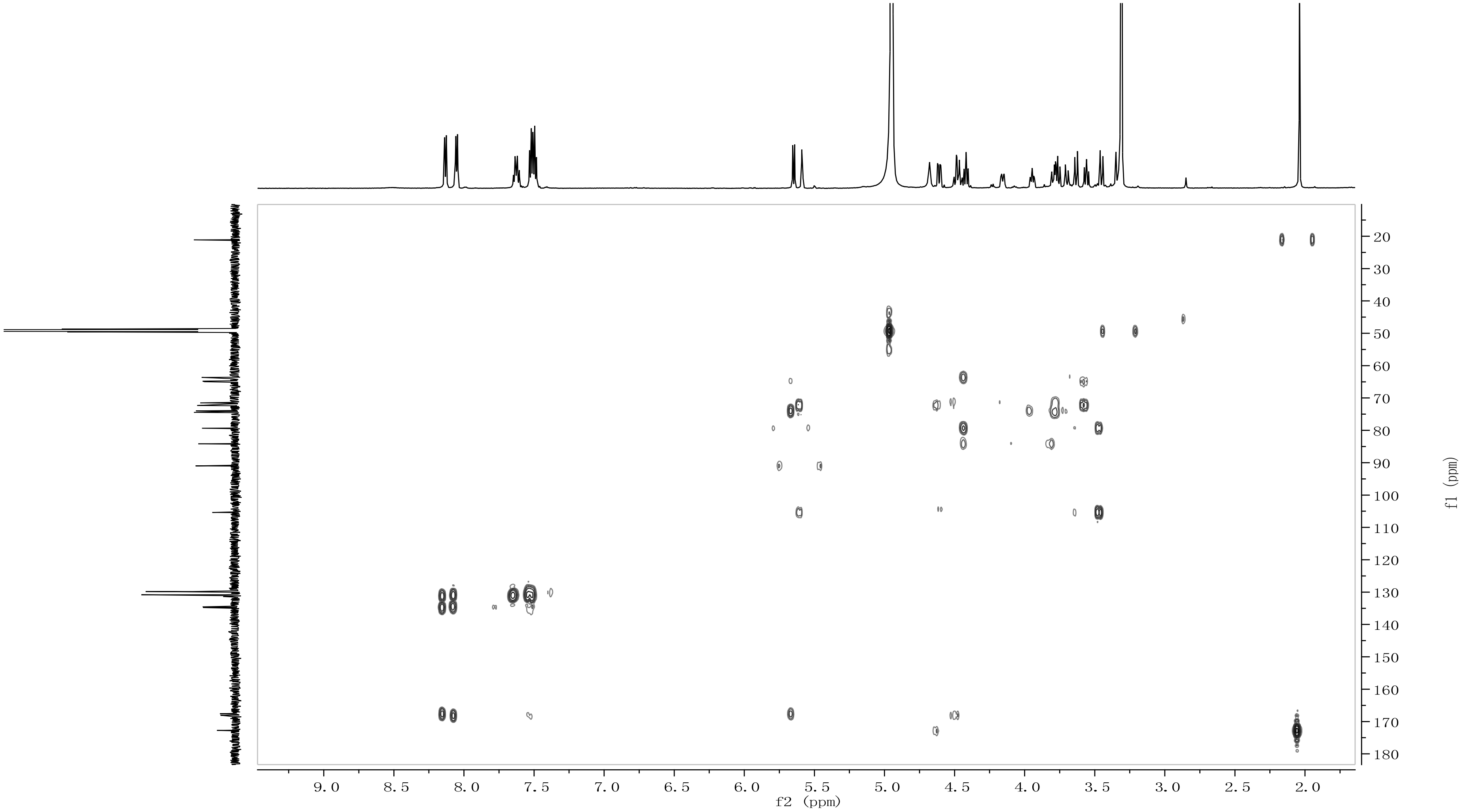

**S12 Optical rotation spectrum of 3,6'-di-*O*-benzoyl-2'-*O*-acetylsucrose (2) in methanol-$d_4$**

Optical rotation measurement

Model : P-1020 (A060460638)

| No. | Sample | Mode | Data | Monitor<br>Blank | Temp.<br>Cell<br>Temp Point | Date<br>Comment<br>Sample Name | Light<br>Filter<br>Operator | Cycle Time<br>Integ Time |
|---|---|---|---|---|---|---|---|---|
| No.1 | 10 (1/3) | Sp.Rot | 32.5000 | 0.0130<br>0.0000 | 22.3<br>50.00<br>Cell | Mon Apr 01 14:28:54 2013<br>0.00080g/mlMeOH<br>YB622EB | Na<br>589nm | 2 sec<br>10 sec |
| No.2 | 10 (2/3) | Sp.Rot | 30.5000 | 0.0122<br>0.0000 | 22.3<br>50.00<br>Cell | Mon Apr 01 14:29:07 2013<br>0.00080g/mlMeOH<br>YB622EB | Na<br>589nm | 2 sec<br>10 sec |
| No.3 | 10 (3/3) | Sp.Rot | 29.7500 | 0.0119<br>0.0000 | 22.3<br>50.00<br>Cell | Mon Apr 01 14:29:21 2013<br>0.00080g/mlMeOH<br>YB622EB | Na<br>589nm | 2 sec<br>10 sec |

+30.9167°

**S13 $^{1}$H NMR spectrum of 3,6'-di-*O*-benzoyl-4'-*O*-acetylsucrose (3) in methanol-*d*$_4$**

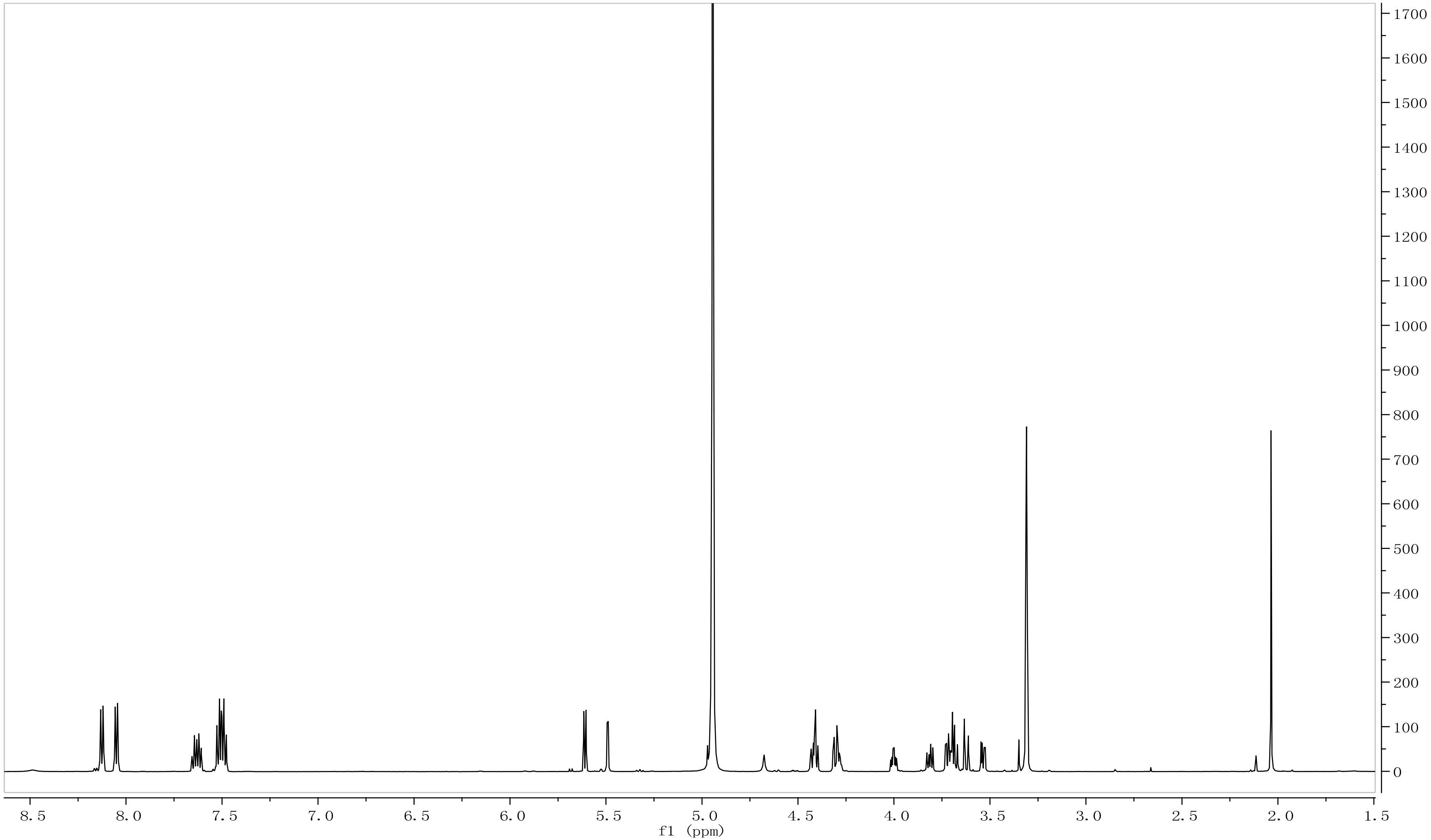

**S14** $^{13}$C NMR (DEPT) spectra of 3,6'-di-*O*-benzoyl-4'-*O*-acetylsucrose (3) in methanol-$d_4$

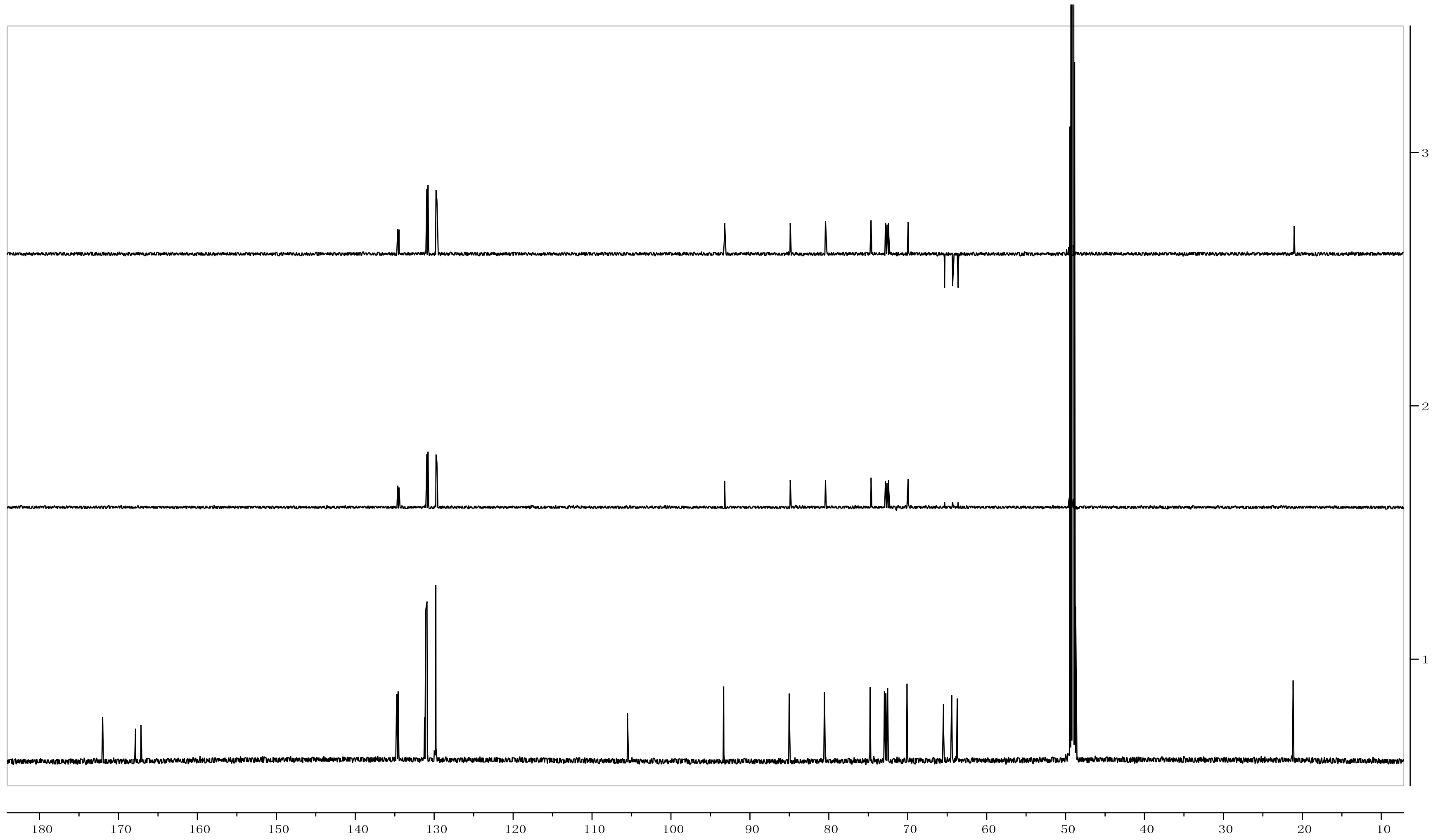

**S15 HSQC spectrum of 3,6'-di-*O*-benzoyl-4'-*O*-acetylsucrose (3) in methanol-$d_4$**

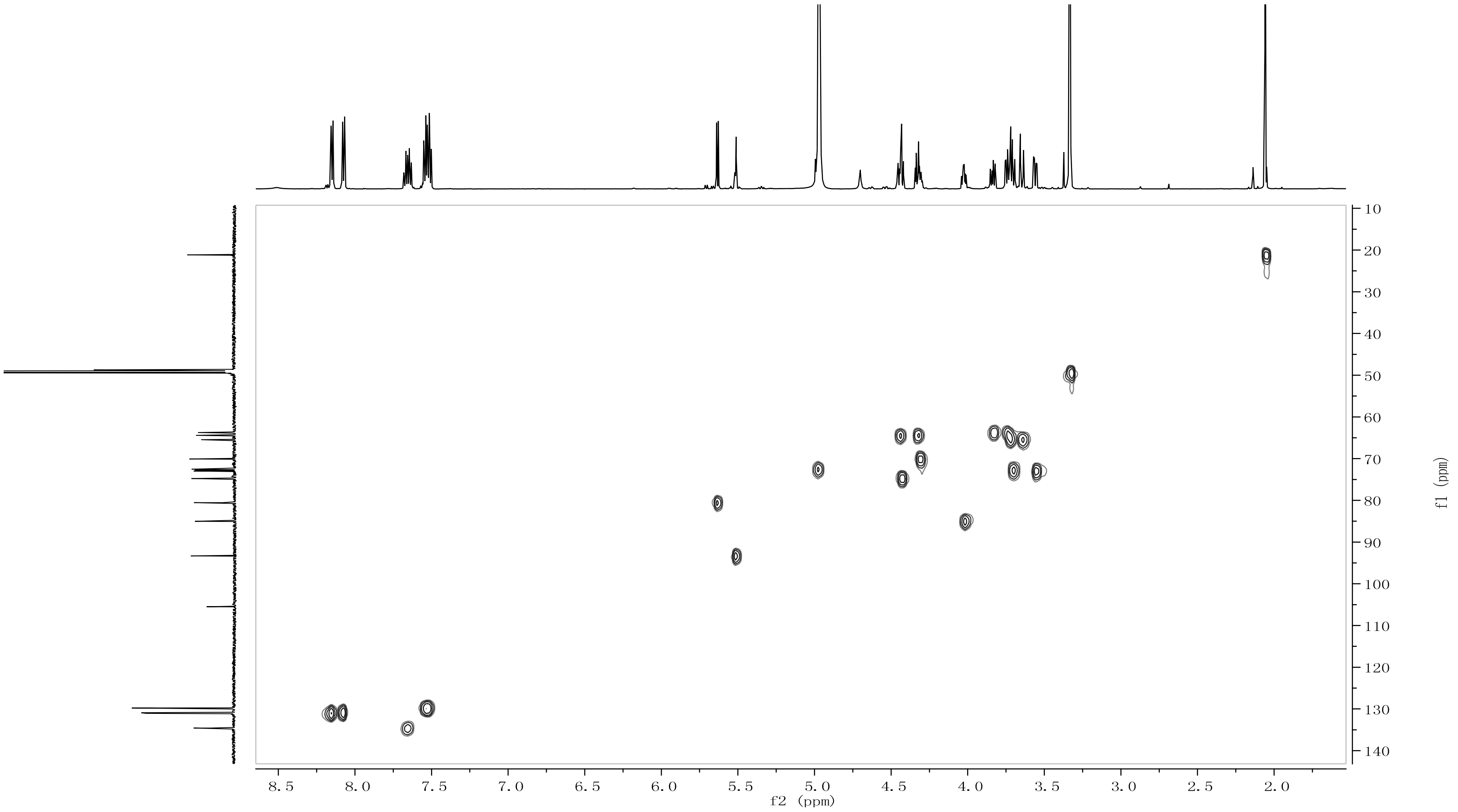

**S16 HMBC spectrum of 3,6'-di-*O*-benzoyl-4'-*O*-acetylsucrose (3) in methanol-$d_4$**

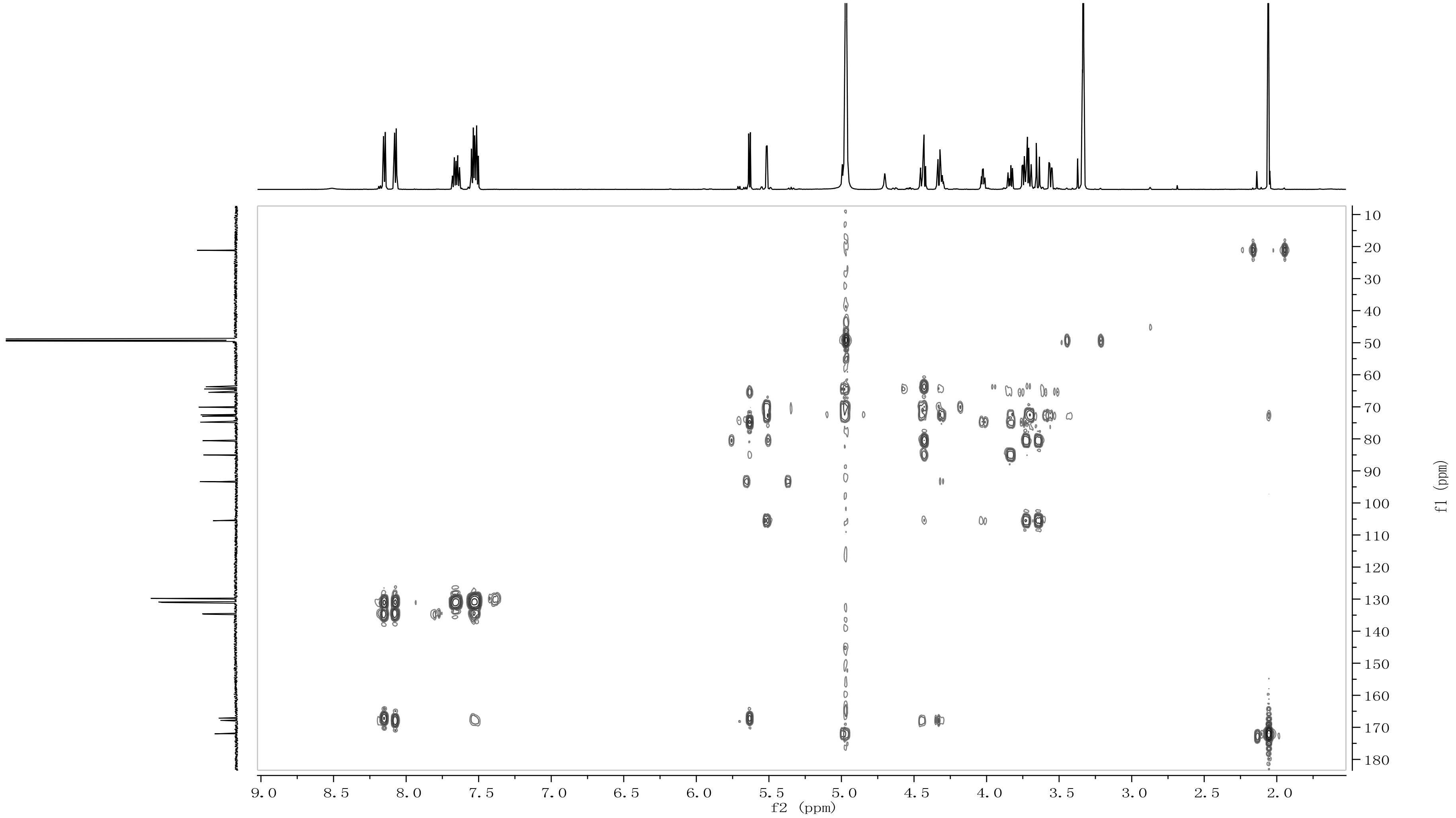

## S17 Optical rotation spectrum of 3,6'-di-*O*-benzoyl-4'-*O*-acetylsucrose (3) in methanol-$d_4$

Optical rotation measurement

Model : P-1020 (A060460638)

| No. | Sample | Mode | Data | Monitor<br>Blank | Temp.<br>Cell<br>Temp Point | Date<br>Comment<br>Sample Name | Light<br>Filter<br>Operator | Cycle Time<br>Integ Time |
|---|---|---|---|---|---|---|---|---|
| No.1 | 12 (1/3) | Sp.Rot | 26.3530 | 0.0224<br>0.0000 | 22.4<br>50.00<br>Cell | Mon Apr 01 14:40:19 2013<br>0.00170g/mlMeOH<br>YB622EC | Na<br>589nm | 2 sec<br>10 sec |
| No.2 | 12 (2/3) | Sp.Rot | 26.3530 | 0.0224<br>0.0000 | 22.4<br>50.00<br>Cell | Mon Apr 01 14:40:33 2013<br>0.00170g/mlMeOH<br>YB622EC | Na<br>589nm | 2 sec<br>10 sec |
| No.3 | 12 (3/3) | Sp.Rot | 25.1760 | 0.0214<br>0.0000 | 22.4<br>50.00<br>Cell | Mon Apr 01 14:40:46 2013<br>0.00170g/mlMeOH<br>YB622EC | Na<br>589nm | 2 sec<br>10 sec |

+25.9608°

**S18 $^1$H NMR spectrum of 3,6'-di-*O*-benzoyl-3'-*O*-acetylsucrose (4) in methanol-$d_4$**

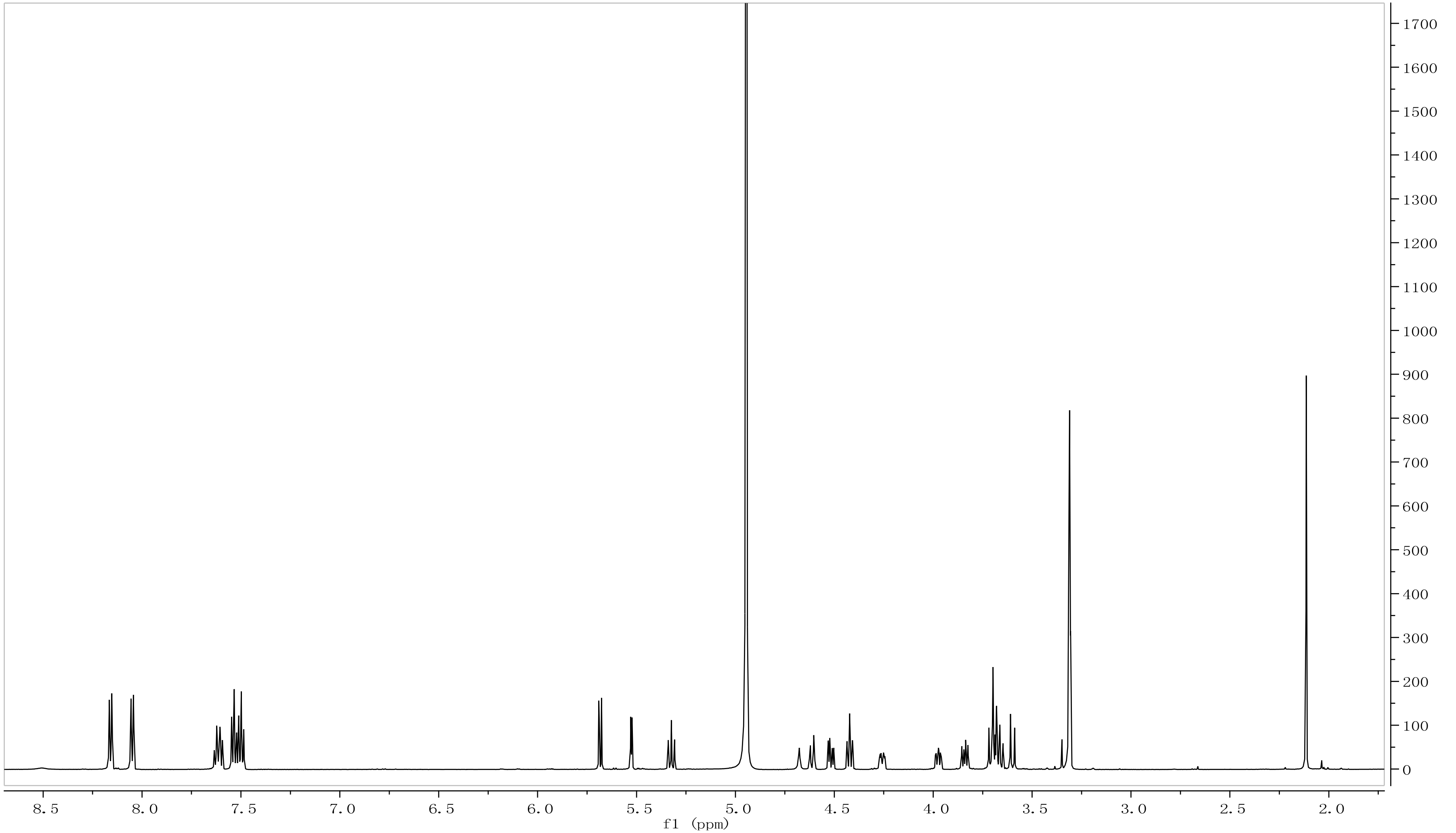

S19 $^{13}$C NMR (DEPT) spectra of 3,6'-di-*O*-benzoyl-3'-*O*-acetylsucrose (4) in methanol-$d_4$

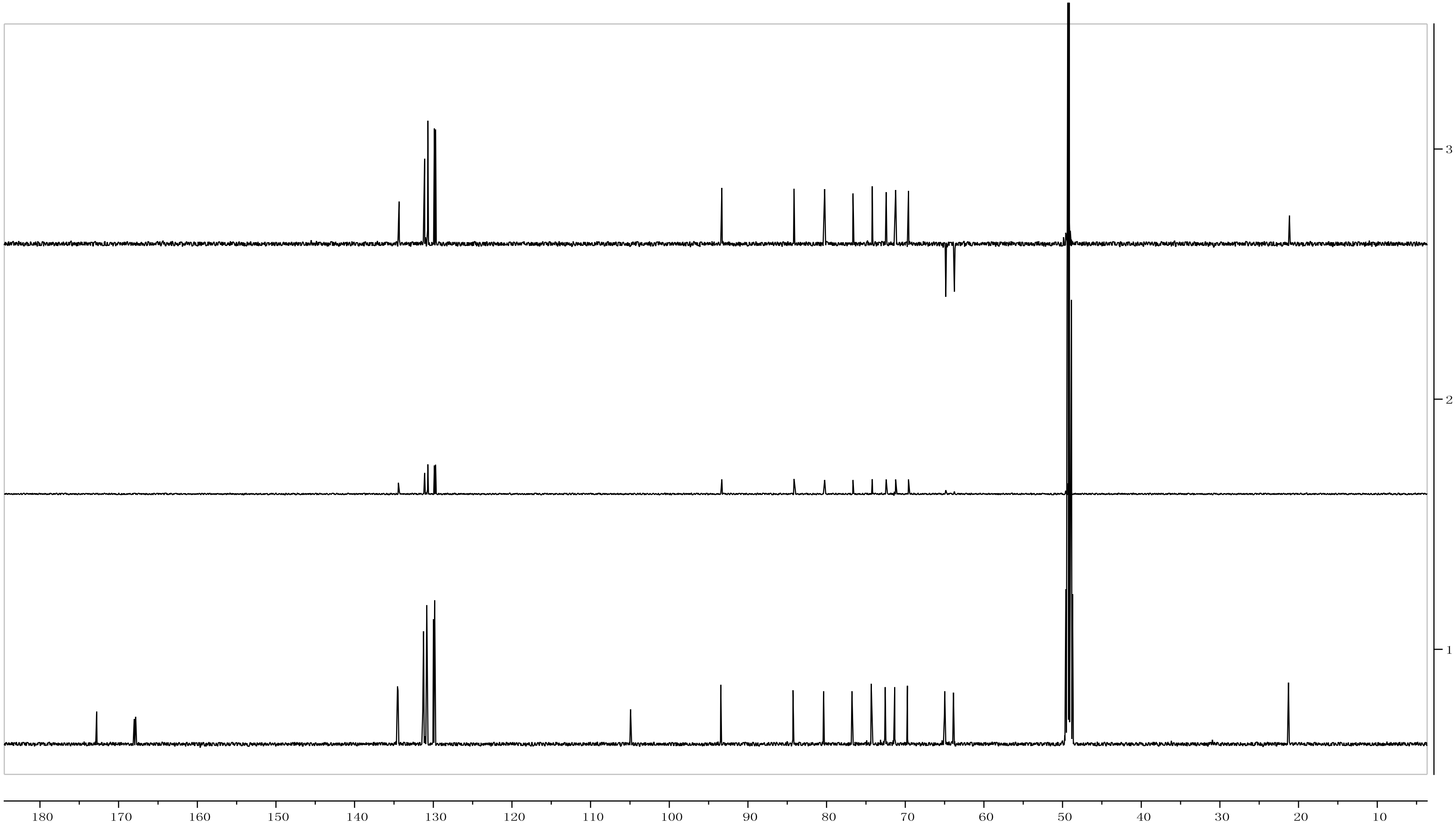

**S20 HSQC spectrum of 3,6'-di-*O*-benzoyl-3'-*O*-acetylsucrose (4) in methanol-$d_4$**

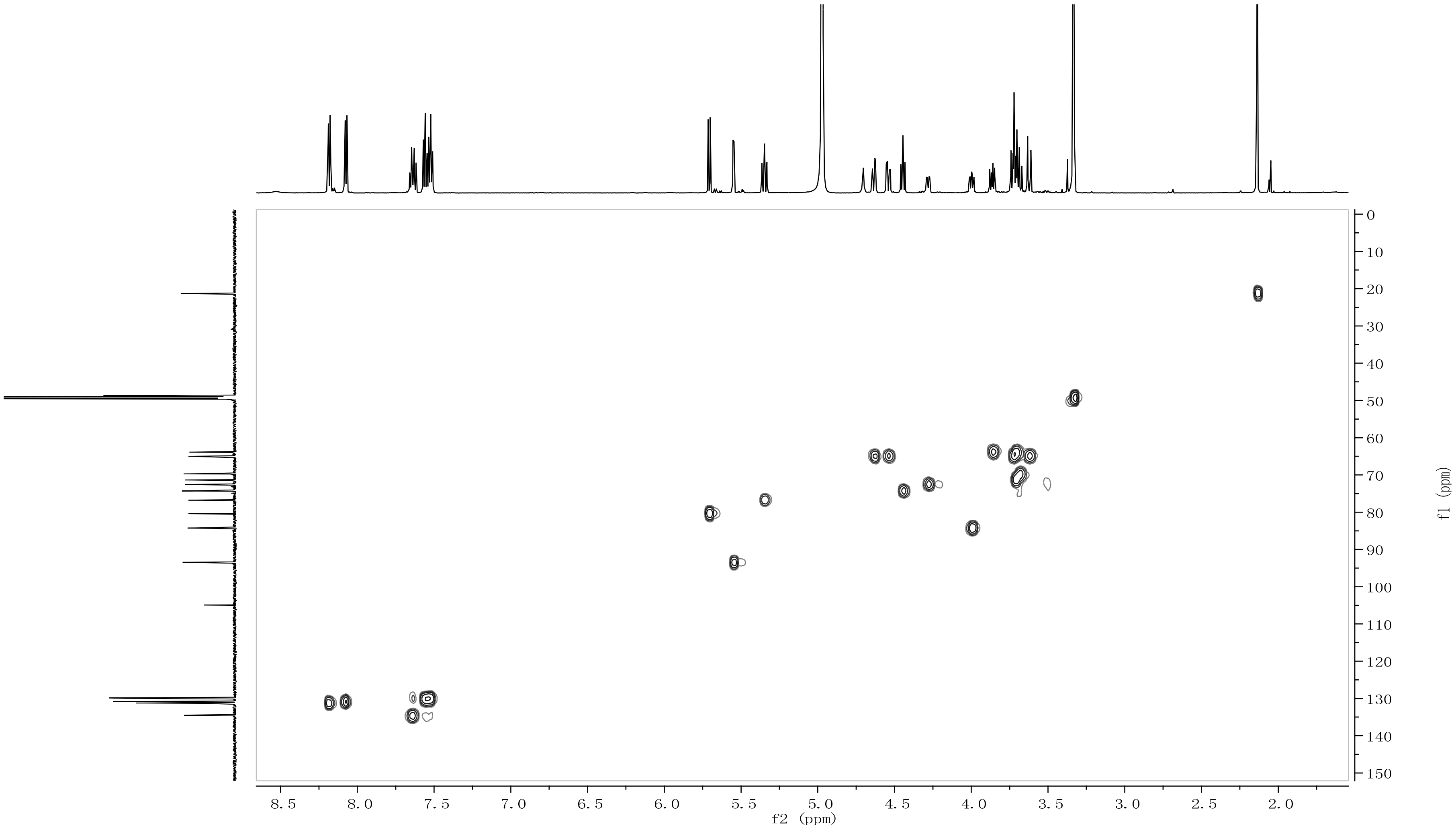

**S21 HMBC spectrum of 3,6'-di-*O*-benzoyl-3'-*O*-acetylsucrose (4) in methanol-$d_4$**

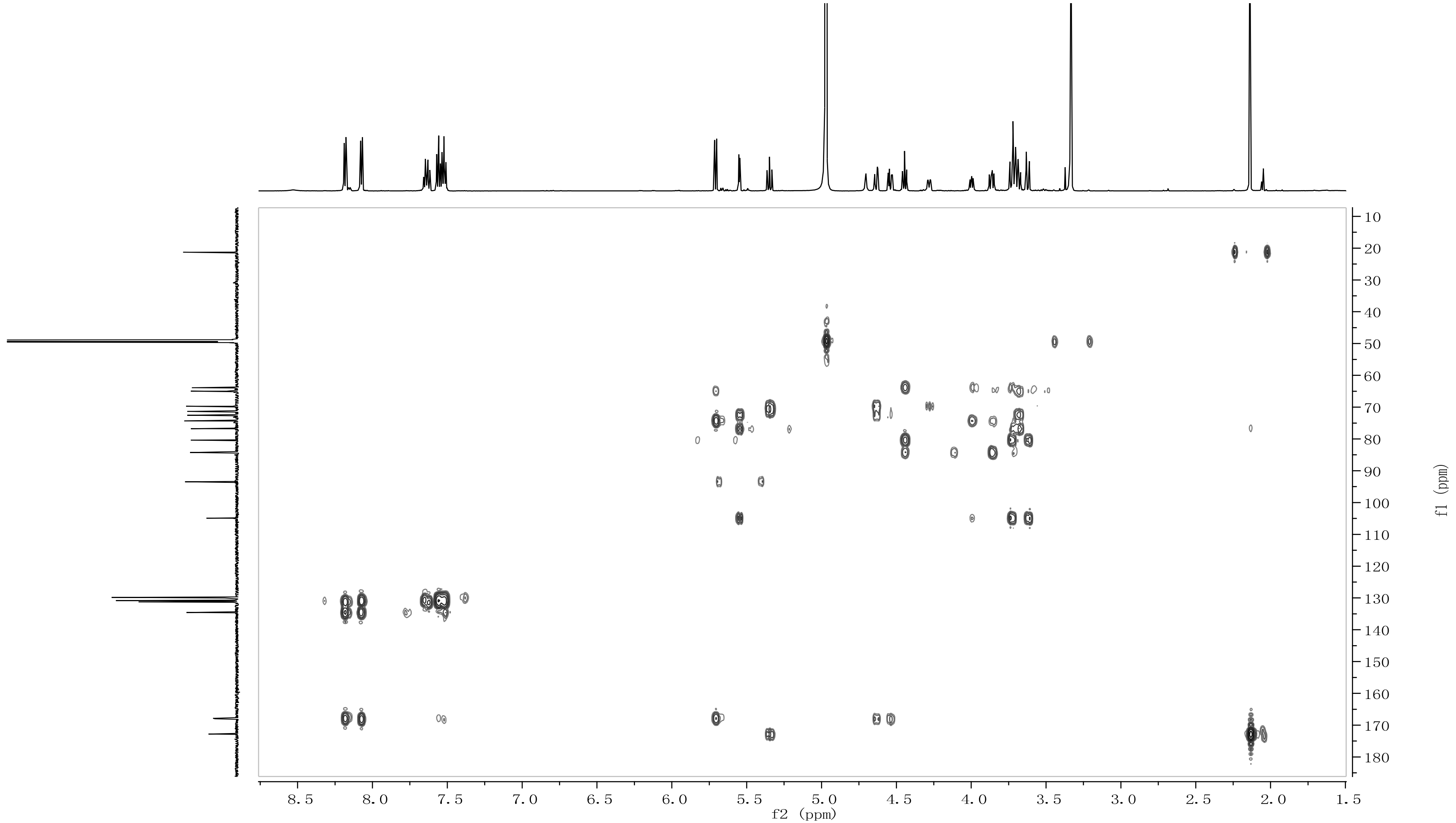

**S22 Optical rotation spectrum of 3,6'-di-*O*-benzoyl-3'-*O*-acetylsucrose (4) in methanol-$d_4$**

Optical rotation measurement

Model : P-1020 (A060460638)

| No. | Sample | Mode | Data | Monitor<br>Blank | Temp.<br>Cell<br>Temp Point | Date<br>Comment<br>Sample Name | Light<br>Filter<br>Operator | Cycle Time<br>Integ Time |
|---|---|---|---|---|---|---|---|---|
| No.1 | 8 (1/3) | Sp.Rot | 26.2860 | 0.0092<br>0.0000 | 22.0<br>50.00<br>Cell | Mon Apr 01 14:00:12 2013<br>0.00070g/mlMeOH<br>YB622ED | Na<br>589nm | 2 sec<br>10 sec |
| No.2 | 8 (2/3) | Sp.Rot | 30.0000 | 0.0105<br>0.0000 | 22.0<br>50.00<br>Cell | Mon Apr 01 14:00:26 2013<br>0.00070g/mlMeOH<br>YB622ED | Na<br>589nm | 2 sec<br>10 sec |
| No.3 | 8 (3/3) | Sp.Rot | 31.7140 | 0.0111<br>0.0000 | 22.1<br>50.00<br>Cell | Mon Apr 01 14:00:39 2013<br>0.00070g/mlMeOH<br>YB622ED | Na<br>589nm | 2 sec<br>10 sec |

+ 29.3333°

**S23 ¹H NMR spectrum of 3-*O*-benzoyl-6'-*O*-(*E*)-cinnamoylsucrose (5) in DMSO-$d_6$**

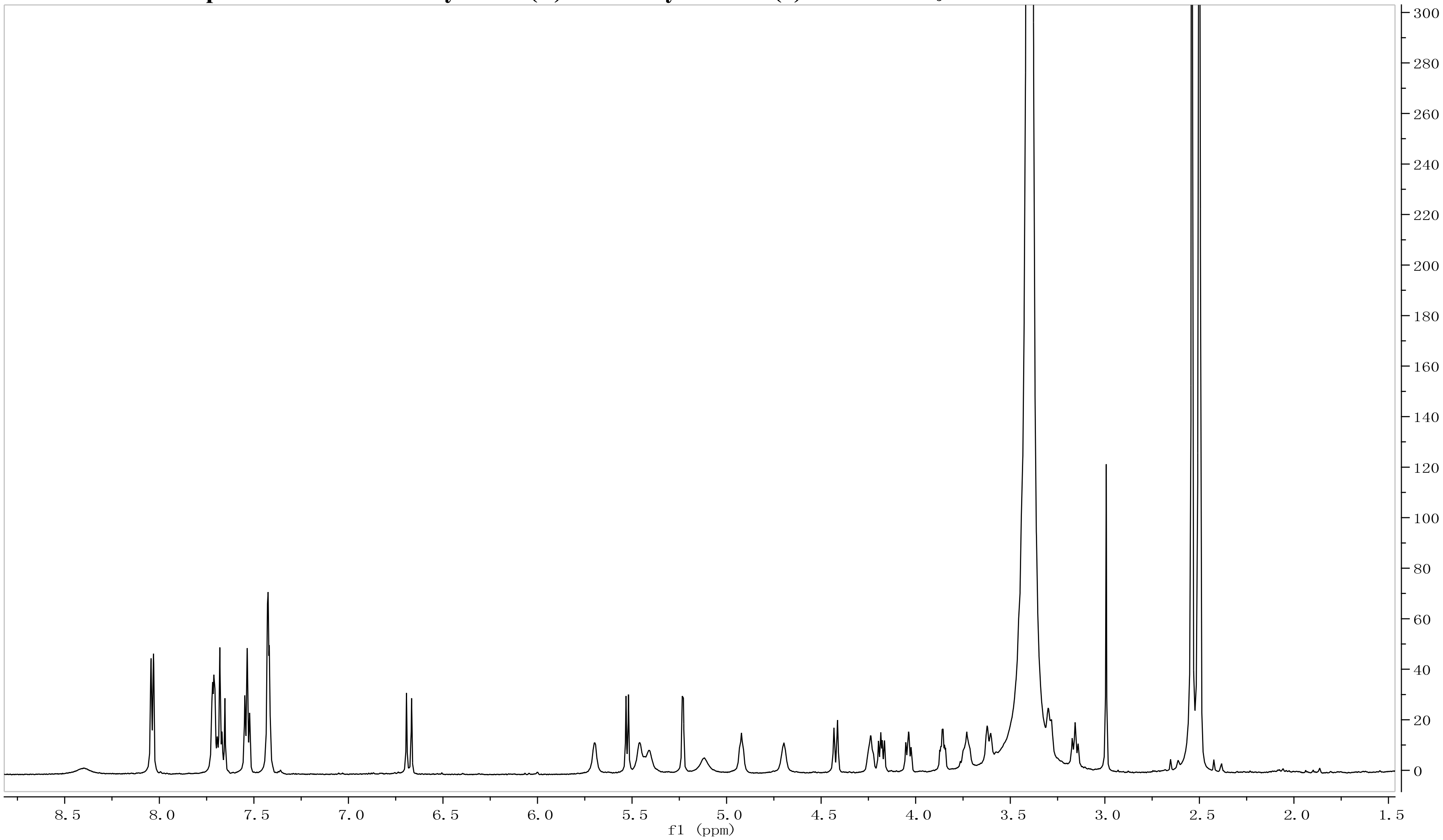

**S24** $^{13}$C NMR (DEPT) spectra of 3-*O*-benzoyl-6'-*O*-(*E*)-cinnamoylsucrose (5) in DMSO-$d_6$

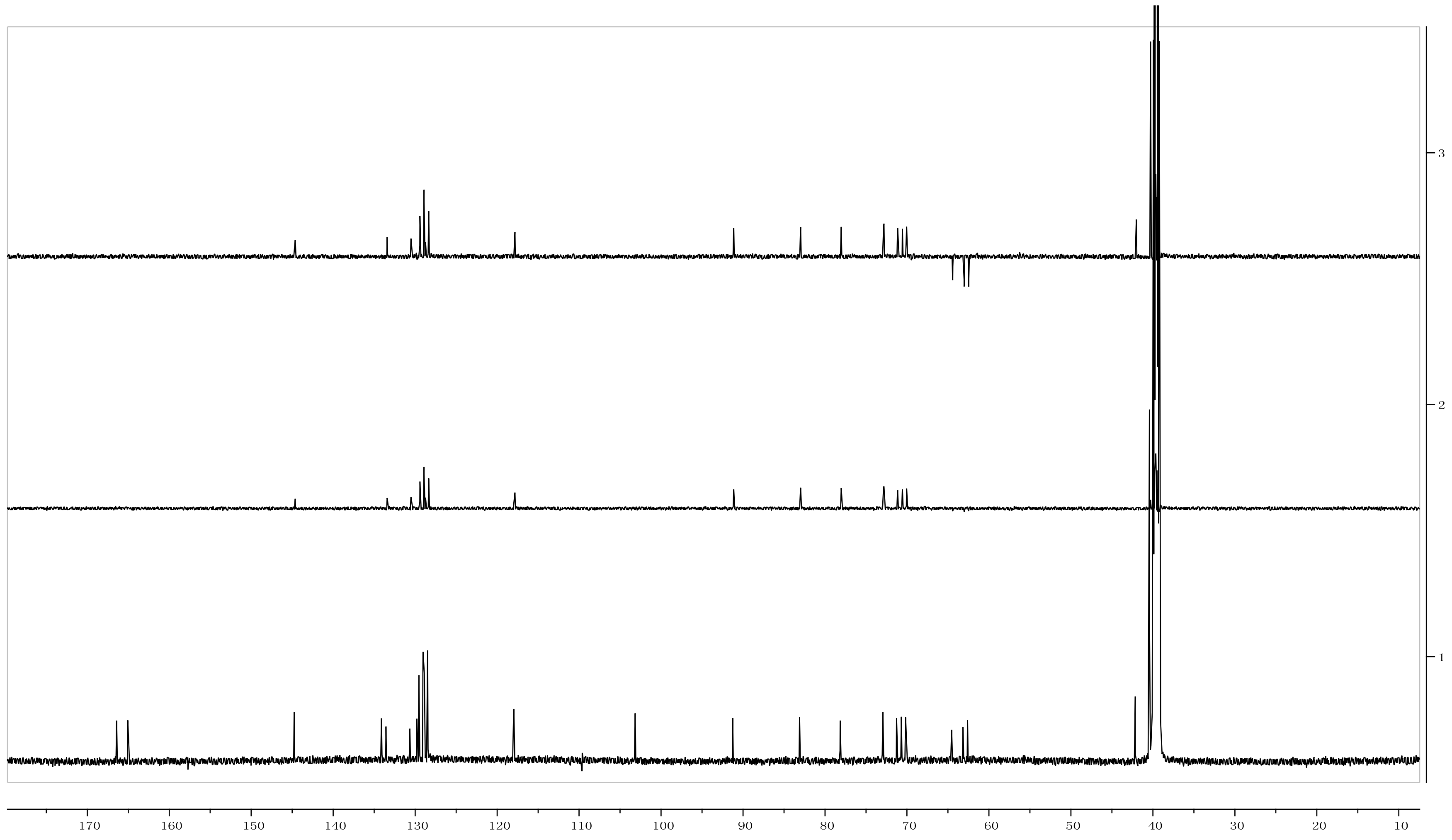

**S25 HSQC spectrum of 3-*O*-benzoyl-6'-*O*-(*E*)-cinnamoylsucrose (5) in DMSO-$d_6$**

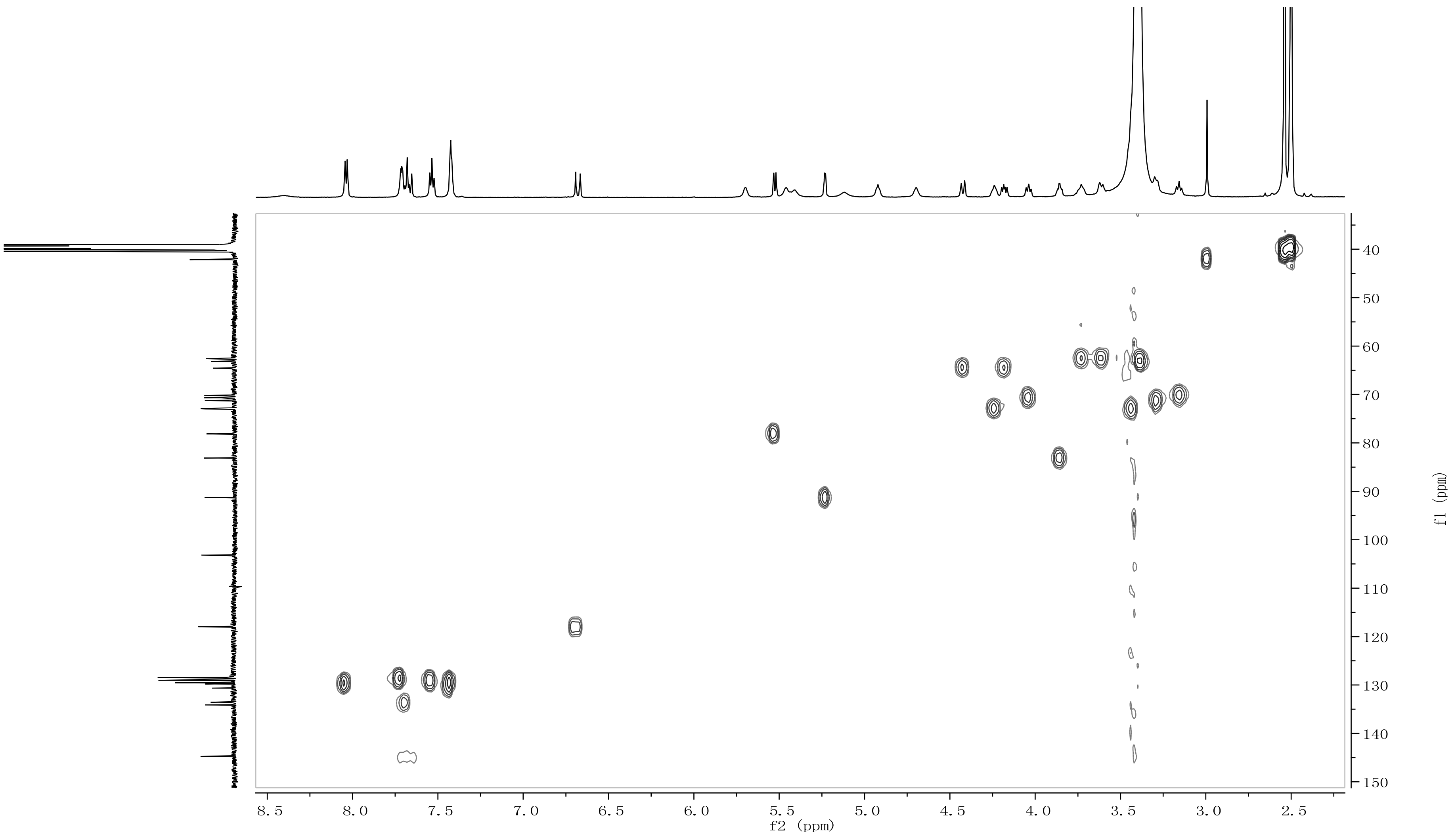

**S26 HMBC spectrum of 3-*O*-benzoyl-6'-*O*-(*E*)-cinnamoylsucrose (5) in DMSO-$d_6$**

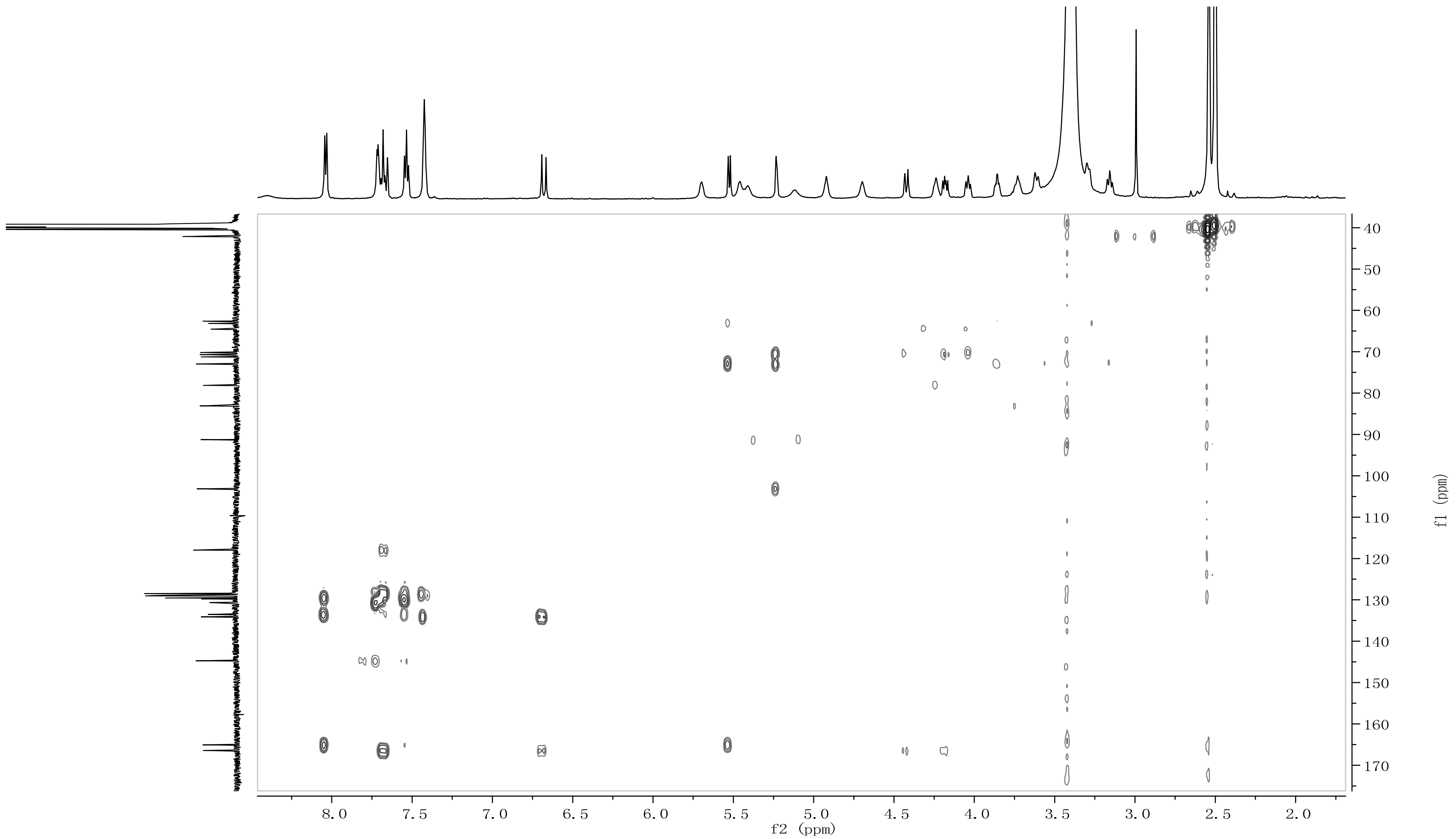

## S27 Optical rotation spectrum of 3-*O*-benzoyl-6'-*O*-(*E*)-cinnamoylsucrose (5) in methanol-$d_4$

Optical rotation measurement

Model : P-1020 (A060460638)

| No. | Sample | Mode | Data | Monitor<br>Blank | Temp.<br>Cell<br>Temp Point | Date<br>Comment<br>Sample Name | Light<br>Filter<br>Operator | Cycle Time<br>Integ Time |
|---|---|---|---|---|---|---|---|---|
| No.1 | 9 (1/3) | Sp.Rot | 24.8570 | 0.0087<br>0.0000 | 22.0<br>50.00<br>Cell | Mon Apr 01 14:07:52 2013<br>0.00070g/mlMeOH<br>YB622DA | Na<br>589nm | 2 sec<br>10 sec |
| No.2 | 9 (2/3) | Sp.Rot | 26.0000 | 0.0091<br>0.0000 | 22.0<br>50.00<br>Cell | Mon Apr 01 14:08:06 2013<br>0.00070g/mlMeOH<br>YB622DA | Na<br>589nm | 2 sec<br>10 sec |
| No.3 | 9 (3/3) | Sp.Rot | 26.0000 | 0.0091<br>0.0000 | 22.0<br>50.00<br>Cell | Mon Apr 01 14:08:19 2013<br>0.00070g/mlMeOH<br>YB622DA | Na<br>589nm | 2 sec<br>10 sec |

+25.6190°
